# Supplementary material for: Optimizing health and nutrition status of migrant construction workers consuming multiple micronutrient fortified rice in Singapore
Source: PLoS One. 2023 Jun 1;18(6):e0285708. doi: 10.1371/journal.pone.0285708 (PMC10234550; doi:10.1371/journal.pone.0285708)
Supplement: S1 Checklist — (PDF) [file pone.0285708.s001.pdf]

TREND Statement Checklist

Paper Section/ Topic	Item No	Descriptor	Reported? ✓	Pg # and line #s
**Title and Abstract**				
Title and Abstract	1	• Information on how unit were allocated to interventions	✓	2
		• Structured abstract recommended	✓	2
		• Information on target population or study sample	✓	2; specific lines # 25-30
Introduction				
Background	2	• Scientific background and explanation of rationale	✓	3-5; specific lines # 95-100
		• Theories used in designing behavioral interventions	n/a	
Methods				
Participants	3	• Eligibility criteria for participants, including criteria at different levels in recruitment/sampling plan (e.g., cities, clinics, subjects)	✓	Male migrant construction workers residing in a single dormitory in Singapore. Pages 6-8
		Method of recruitment (e.g., referral, self-selection), including the sampling method if a systematic sampling plan was implemented	✓	Selection based on clinical examination and health interview. Pages 6-8
		Recruitment setting	✓	6; specific lines # 109-112
		Settings and locations where the data were collected	✓	6-8;
Interventions	4	• Details of the interventions intended for each study condition and how and when they were actually administered, specifically including:		
		○ Content: what was given?	✓	Multiple micronutrient fortified rice; page 8-9; line # 17~~7~~0-18~~2~~9
		○ Delivery method: how was the content given?	✓	Meals with fortified rice were catered to the workers; page 6
		○ Unit of delivery: how were the subjects grouped during delivery?	✓	Individual meals were delivered to each worker; page 6; line# 114-119
		○ Deliverer: who delivered the intervention?	✓	Meals were delivered by designated and licensed caterer; page 6; line# 114-115
		○ Setting: where was the intervention delivered?	✓	Meals were consumed at the construction site and at the dormitory in evenings; page 6;

TREND Statement Checklist

				line# 115-117
		○ Exposure quantity and duration: how many sessions or episodes or events were intended to be delivered? How long were they intended to last?	✓	The intervention of providing multiple fortified rice lasted 6 months; page 6; specific line# 12~~3~~5-12~~7~~8
		○ Time span: how long was it intended to take to deliver the intervention to each unit?	✓	Delivery was daily with one delivery in the morning and one in the evening; page 6; specific line# 115-117
		○ Activities to increase compliance or adherence (e.g., incentives)	✓	Page 6/7; line# 130-13~~2~~3
Objectives	5	Specific objectives and hypotheses	✓	Page 7; specific line# 143~~37~~-14~~2~~8<br>Don’t think we spill out the hypotheses in the article<br>Except: page4-5; line# 82-100
Outcomes	6	Clearly defined primary and secondary outcome measures	✓	Page 7; specific line# 143~~37~~-14~~2~~8
		Methods used to collect data and any methods used to enhance the quality of measurements	✓	Health and anthropometric measurements were done in line with accepted practices described by WHO and under supervision of medical trained staff where needed; international accepted equipment was used to perform different measurements; page 7; specific lines # 14~~3~~9-163~~57~~ and 192~~85~~-206~~199~~.
		Information on validated instruments such as psychometric and biometric properties	✓	Specific equipment for anthropometry; page 7; specific lines# 1~~45~~51-1~~47~~53
Sample Size	7	How sample size was determined and, when applicable, explanation of any interim analyses and stopping rules	✓	A sample size of 180 participants with a maximum anticipated subject attrition of 22% was estimated based on the following: a paired t-test, a power of 80% to achieve

TREND Statement Checklist

		• • •		significance on all primary endpoints simultaneously (95% power for each primary endpoint individually) and a two-sided alpha of 5% (overall), and an effect size of ≥0.30 for all parameters. The study suffered from high dropout rates; first, with 40 of the original 180 workers who did not report for screening, and second, an additional 40 of the 140 remaining participants who either left the study or declined involvement in the final study visit. The aimed sample size of 140 for an overall power of 80% was not achieved, and therefore, the study might be underpowered to pick up significant effects.
Assignment Method	8	Unit of assignment (the unit being assigned to study condition, e.g., individual, group, community)	✓	Individual male migrant workers received meals with fortified rice; page6; line# 120-127

•

		Method used to assign units to study conditions, including details of any restriction (e.g., blocking, stratification, minimization)	✓	The study subjects had to pass the study enrollment criteria which were based on a set of conditions such as a certain BMI range, healthy – based on health questionnaire, clinical examination and blood pressure and anthropometric measurements. Page 7; specific line# 149-165
		Inclusion of aspects employed to help minimize potential bias induced due to non-randomization (e.g., matching)<br>•<br>•	✓	Subjects were not enrolled if not passing the enrollment criteria; page 6-8;
Blinding (masking)	9	• Whether or not participants, those administering the interventions, and those assessing the outcomes were blinded to study condition assignment; if so, statement regarding how the blinding was accomplished and how it was assessed.	No	This is a non-randomized and not controlled study; all participants were aware of the study conditions; page 6;
Unit of Analysis	10	• Description of the smallest unit that is being analyzed to assess intervention effects (e.g., individual, group, or community)	✓	The smallest group being analyzed is 7 subjects, as we ae looking at changes in homocysteine levels derived from Indian subjects; page 13; Table 2; line#261
		• If the unit of analysis differs from the unit of assignment, the analytical method used to account for this (e.g., adjusting the standard error estimates by the design effect or using multilevel analysis)	n/a	
Statistical Methods	11	• Statistical methods used to compare study groups for primary methods outcome(s), including complex methods of correlated data	✓	One-sample and two sample t-test; page 10; line#223-225
		• Statistical methods used for additional analyses, such as a subgroup analyses and adjusted analysis	✓	One-sample and two sample t-test; page 10; line#223-225
		Methods for imputing missing data, if used	n/a	
		Statistical software or programs used	✓	SPSS 26 software (IBM Corp., Released 2019, Armonk, NY, USA) was

TREND Statement Checklist

				used for all analyses; p 10; line# 219-220
**Results**				
Participant flow	12	Flow of participants through each stage of the study: enrollment, assignment, allocation, and intervention exposure, follow-up, analysis (a diagram is strongly recommended)	✓	The below items are captured in the Participant Flow Chart Figure 1
		○ Enrollment: the numbers of participants screened for eligibility, found to be eligible or not eligible, declined to be enrolled, and enrolled in the study	✓	Flow Chart Figure 1; page 11; specific line# 233-234
		○ Assignment: the numbers of participants assigned to a study condition	✓	Described in Table 1; page 11-12; specific line# 242-245
		○ Allocation and intervention exposure: the number of participants assigned to each study condition and the number of participants who received each intervention	✓	Page 12; specific line# 246-252
		○ Follow-up: the number of participants who completed the follow-up or did not complete the follow-up (i.e., lost to follow-up), by study condition	✓	Description of subjects who took home leave during the 6 months intervention; page 13; line# 246-252; also supplementary table 3a and 3b
		○ Analysis: the number of participants included in or excluded from the main analysis, by study condition	✓	All participants with both base- and endline measurements were included in the study; Table 2; page 13
		• Description of protocol deviations from study as planned, along with reasons	✓	Sample size line#120-122; Quantity of rice Line#129-130; Incentives line#130-132; inclusion criteria (age) line# 163-164; statistical analysis (intention-to-treat) line# 226-227; Homocysteine sub-sample size line#252-254
Recruitment	13	Dates defining the periods of recruitment and follow-up	✓	03-2018 to 06-2018; see online registry NCT04343508; page 7; line#134-135
Baseline Data	14	Baseline demographic and clinical characteristics of participants in each study condition	✓	Description of baseline demographics page 11 ; line#234-240; Table 1

		Baseline characteristics for each study condition relevant to specific disease prevention research	✓	Healthy workeers were enrolled in the study – description of inclusion and exclusion criteria page 7-8; line#153-158
		Baseline comparisons of those lost to follow-up and those retained, overall and by study condition	✓	See figure 1 flowchart page 12; line#241
		Comparison between study population at baseline and target population of interest	N/a	
Baseline equivalence	15	• Data on study group equivalence at baseline and statistical methods used to control for baseline differences	n/a	
Numbers analyzed	16	• Number of participants (denominator) included in each analysis for each study condition, particularly when the denominators change for different outcomes; statement of the results in absolute numbers when feasible	✓	Table 2; page 13
		• Indication of whether the analysis strategy was "intention to treat" or, if not, description of how non-compliers were treated in the analyses	✓	Intention to treat line# 226-227
Outcomes and estimation	17	• For each primary and secondary outcome, a summary of results for each estimation study condition, and the estimated effect size and a confidence interval to indicate the precision	✓	P-values, mean, and mean changes are given instead of confidence intervals; page 13; table 2
		• Inclusion of null and negative findings	✓	All data is presented including those data that were proven not statistically significant; page 13; table 2
		Inclusion of results from testing pre-specified causal pathways through which the intervention was intended to operate, if any	n/a	
Ancillary analyses	18	• Summary of other analyses performed, including subgroup or restricted analyses, indicating which are pre-specified or exploratory	n/a	
Adverse events	19	• Summary of all important adverse events or unintended effects in each study condition (including summary measures, effect size estimates, and confidence intervals)	✓	Data was collected on AEs and is described in the Methods and Results section of the manuscript; line# 263-267; 299-304
DISCUSSION				
Interpretation	20	Interpretation of the results, taking into account study hypotheses, sources of potential bias, imprecision of measures, multiplicative analyses, and other limitations or weaknesses of the study	✓	Page 15-17; specific lines to strengths and limitations of the study are in line# 358-368

		• Discussion of results taking into account the mechanism by which the intervention was intended to work (causal pathways) or alternative mechanisms or explanations	✓	Pages 15-17; specific line# 309-324;
		Discussion of the success of and barriers to implementing the intervention, fidelity of implementation	✓	Pages 15-17; specific line# 325-357
		Discussion of research, programmatic, or policy implications	✓	Page 18; specific line# 370-383
Generalizability	21	Generalizability (external validity) of the trial findings, taking into account the study population, the characteristics of the intervention, length of follow-up, incentives, compliance rates, specific sites/settings involved in the study, and other contextual issues	✓	Pages 15-19; line# 284-383
Overall Evidence	22	• General interpretation of the results in the context of current evidence and current theory	✓	Page 15; specific line# 284-293 and 353-357

From: Des Jarlais, D. C., Lyles, C., Crepaz, N., & the Trend Group (2004). Improving the reporting quality of nonrandomized evaluations of behavioral and public health interventions: The TREND statement. *American Journal of Public Health*, 94, 361-366. For more information, visit: http://www.cdc.gov/trendstatement/

•
