# Supplementary material for: Optimizing health and nutrition status of migrant construction workers consuming multiple micronutrient fortified rice in Singapore
Source: PLoS One. 2023 Jun 1;18(6):e0285708. doi: 10.1371/journal.pone.0285708 (PMC10234550; doi:10.1371/journal.pone.0285708)
Supplement: S1 Table — (PDF) [file pone.0285708.s002.pdf]

Supplementary Table 1

Proposed Menu For Bangala Food
Monday to Sunday

| DAY | Break Fast | Lunch | Dinner | Remarks |
|---|---|---|---|---|
| Monday | Parta/Chapthi x 02 pcs<br>Chana dhal or Sugi or Bhaji | Masoor Dhal<br>Fish curry With Vegetables<br>Rice | Masoor Dhal<br>Bhaji or Botah<br>Mutton curry<br>Rice | Mutton curry can be replaced by Chicken Curry or Egg curry or Fish Curry due to religious or other reasons |
| Tuesday | Parta/Chapthi x 02 pcs<br>Chana dhal or Sugi or Bhaji | Masoor Dhal<br>Fish curry With Vegetables<br>Rice | Masoor Dhal<br>Bhaji or Botah<br>Beef curry<br>Rice | Beef curry can be replaced by Chicken Curry or Egg curry or Fish Curry due to religious or other reasons |
| Wednesday | Parta/Chapthi x 02 pcs<br>Chana dhal or Sugi or Bhaji | Masoor Dhal<br>Fish curry With Vegetables<br>Rice | Masoor Dhal<br>Bhaji or Botah<br>Chicken curry<br>Rice | |
| Thursday | Parta/Chapthi x 02 pcs<br>Chana dhal or Sugi or Bhaji | Masoor Dhal<br>Fish curry With Vegetables<br>Rice | Masoor Dhal<br>Bhaji or Botah<br>Beef curry<br>Rice | Beef curry can be replaced by Chicken Curry or Egg curry or Fish Curry due to religious or other reasons |
| Friday | Parta/Chapthi x 02 pcs<br>Chana dhal or Sugi or Bhaji | Masoor Dhal<br>Fish curry With Vegetables<br>Rice | Masoor Dhal<br>Bhaji or Botah<br>Mutton curry<br>Rice | Mutton curry can be replaced by Chicken Curry or Egg curry or Fish Curry due to religious or other reasons |
| Saturday | Parta/Chapthi x 02 pcs<br>Chana dhal or Sugi or Bhaji | Masoor Dhal<br>Fish curry With Vegetables<br>Rice | Masoor Dhal<br>Bhaji or Botah<br>Chickencurry<br>Rice | |
| Sunday | Parta/Chapthi x 02 pcs<br>Chana dhal or Sugi or Bhaji | Masoor Dhal<br>Fish curry With Vegetables<br>Rice | Masoor Dhal<br>Bhaji or Botah<br>Beef curry<br>Rice | Beef curry can be replaced by Chicken Curry or Egg curry or Fish Curry due to religious or other reasons |

Note :
The breakfast menu will be differ upon the availability
To the best we will follow the above menu but depends upon the availability and stock of the ingredient.
There may be some changes in the above menu then and there
