# Supplementary material for: Optimizing health and nutrition status of migrant construction workers consuming multiple micronutrient fortified rice in Singapore
Source: PLoS One. 2023 Jun 1;18(6):e0285708. doi: 10.1371/journal.pone.0285708 (PMC10234550; doi:10.1371/journal.pone.0285708)
Supplement: S2 Table — (PDF) [file pone.0285708.s003.pdf]

Supplementary Table 2

Proposed Menu For Indian Food			
Sunday to Saturday			
DAY	BreakFast	Lunch	Dinner
Sunday Non Veg	Chappa ti Kurma	Nandu Curry Muttai Samba! Rice+Butter Milk	Karuvattu Curry Chan na Masai Rice+Rasam
Sunday Veg	Chappati Kurma	Vegetable Ku nua +Channa Masala +Rice+Butter Milk	Sambar + Moormelagai Veg Masala Paratal Spicy Rice + Rsam
Monday Non Veg	Roti prata + **Kuruma**	Fish Cu rry + Fish Fry + Rice +Butter Milk	Chicken Curry + Veg Rice + Rasam
Monday Veg	Roti prata + Kuruma	Vathal Curry +Potato Fry / Biter Gourd+ Rice + Butter Milk	Sambar + Veg Moormelagai / Appalam Rice Rasam
Tuesday Veg	Pulli Rice	Pulli Curry Cabbage / Saraka Saraka / Cabbage Kottu Rice+Butter Milk	Sambar + Rava Palpayasam Potato Green Peas Paratal Rice + Rasam
Wednesday Non Veg	Chappati Kurma	Fish Cu rry + Fish Fry +Rice +Butter Milk	Pepper Chicken Chicken Dalcha Rice+Rasam
Wednesday Veg	Chappati Kurma	Moor Curry Ca uliflower Fry Moormelagai Rice+Butter Milk	Sambar + Cabbage Kuttu Moormelagai / Appalam + Rice Rasam
Thursday Non Veg	Ediyappam Sugar	Chicken Fry + Sambar + Rice+ Butter Milk	Mutton Curry + Mutton Masala+Veg+Rice+ Rasam
Thursday Veg	Ediyappam Sugar	Samba r+Moormelagai Pakoda / Bitter Gourd fry Rice + Butter Milk	Dhall Thaduka + Califlower + Potato Masala Paratal Rice + Rasam
Friday	Lemon Rice	Moor Cu rry Pnli Ma nthi(brin jal, Vendi, Pottato,Mochai) Pa ratalMoormelagai / AppalamRice+ Rasam	Sambar+Potato Masala Paratal Kesa ri / Pa ruppn Payasam / Yellow Rava paysam / Rice + Rasam
Saturday Non Veg	Dhosai / Idly + Dhall	Fish Curry + Fish Fry Rice+Butter Milk	Briyaui Rice Chicken Cu rry/K uru ma Raitha Salad (Cumcumber,Oninon, Yogurt)
Saturday Veg	Dhosai / Id ly + Dhall	Puli Cu rry + Brinjal Fry + Rice +Butter Milk	Veg Briyani Vegetable Kurma + Brinjal Fry+ Raitha Salad (Cumcumber, Onion, Carrot, Yogurt)
