# Supplementary material for: Optimizing health and nutrition status of migrant construction workers consuming multiple micronutrient fortified rice in Singapore
Source: PLoS One. 2023 Jun 1;18(6):e0285708. doi: 10.1371/journal.pone.0285708 (PMC10234550; doi:10.1371/journal.pone.0285708)
Supplement: S3 Table — a. Total number of workers on home leave. * subjects do have both baseline and post-intervention. b. Weekly distribution of workers who took home leave. * subjects do have both baseline and post-intervention. (ZIP) [file pone.0285708.s004.zip › S3b Table.pdf]

Weeks of home leave	subjects enrolled in the study*	Percent (%)
0	86	86
2	1	1
4	4	4
5	2	2
6	2	2
7	1	1
8	4	4
Total	100	100

* subjects do have both baseline and endline endpoints
