# Supplementary material for: Optimizing health and nutrition status of migrant construction workers consuming multiple micronutrient fortified rice in Singapore
Source: PLoS One. 2023 Jun 1;18(6):e0285708. doi: 10.1371/journal.pone.0285708 (PMC10234550; doi:10.1371/journal.pone.0285708)
Supplement: S1 File — a English version of health questionnaire. b Tamil version of health questionnaire. c Bengali version of health questionnaire. (ZIP) [file pone.0285708.s005.zip › S4a English version of health questionnaire.pdf]

VISIT 1, ENROLLEMENT

Date of Enrollment visit: ___ ___ ___ **(dd/mm/yyyy)**

Name of research nurse completing the form: ______________________________

INFORMED CONSENT SIGNATURE

Did the subject sign the informed consent?

☐ **YES** ☐ **NO**

Informed consent obtained: ___ ___ ___ **(dd/mm/yyyy)**

DEMOGRAPHICS AND ANTHROPOMETRY (ENROLLMENT)

**Date of birth**	(dd/mm/yyyy)
**Age (years; Subjects 21-50 years)**	
**Ethnic groups**	
**Height (cm)**	
**Weight (kg)**	
**BMI (kg/m2)**	.
**Education (highest level completed)**	☐ **<High School graduate**<br>☐ **High School graduate**<br>☐ **College Diploma**
**Previous Occupation**	

MEDICAL HISTORY

(ENROLLMENT)

Does the subject have a clinically significant history of any of the following?				
(*Check the corresponding box*)<br>**NO**	**YES**	**DOES NOT KNOW**		If checked **YES,**<br>Please specify the start date and condition below. If there is any medication taken on these conditions, please specify in Appendix 5.
☐	☐	☐	Gastrointestinal (ulcers, dyspepsia)	
☐	☐	☐	Eating Disorder	
☐	☐	☐	Renal disease	
☐	☐	☐	Chronic liver disease	
☐	☐	☐	Cardiovascular disease	
☐	☐	☐	Hematologic disorder	
☐	☐	☐	Infectious disease	
☐	☐	☐	Neurological	
☐	☐	☐	Recent surgery (within 1 year)	

Project Code No. :	SUBJECTINITIALS SUBJECT SCREENING NO	SUBJECTNUMBER

CRF—OBVO0104DSM _19042017

DIETARY SUPPLEMENT & RICE CONSUMPTION QUESTIONNAIRE
(ENROLLMENT BASELINE)

1. Have you used or taken any vitamins, minerals, herbal products or other dietary supplements in the last ONE MONTH?

☐ **YES**

☐ **NO**

If **YES,** please specify the dietary supplements,the name and/ or the brand/ manufacturer.

Here are some examples of the dietary supplement:

- **Vitamins:** Multivitamins, Vitamin A/B/C/ D/ E, Beta Carotene, Folic acid, Niacin, etc
- **Minerals:** Multiminerals**,** Calcium, Magnesium, Chromium, Iron, Potassium, Selenium, Zinc
- **Herbs/ botanicals:** Echinacea, Garlic, Kava, St John's wort, Grape, Saw palmetto

Name of Supplement	Frequency	Start Date (DD/MM/YY)	Ongoing	If not Ongoing- End date (DD/MM/YY)
			☐YES☐NO	

2. How many times a week do you consume **RICE**?
   A. More than 14 meals
   B. Less than 14 meals - continue with question 3

3. If subject eats **LESS THAN 14 MEALS of rice** a week, please answer:
   What type food do you take?
   A. Bread/Chapati/Pratha/Kulcha/Phulka
   B. Noodles/Rava idli
   C. Others, please specify……………………………………………………………………………………

Investigator:________________________ Monitored by:__________________________

(Initial & signature) (Initial & signature)

DATE: ☐☐ ☐☐ ☐☐☐☐ ☐☐ ☐☐ ☐☐☐☐
(dd/mm/yyyy)

VISIT 1, SUMMARY

Date of Visit 1: ☐☐ ☐☐ ☐☐☐☐ **(dd/mm/yyyy)**

Name of research nurse/CPM completing the form: ______________________________

Please Check off when tasks (Visit 1 baseline) done:

TASKS:

☐ PI provide protocol information including reporting of adverse events (AEs) that may arise to potential subjects. ICF briefing session.

☐ Detailed Medical History

☐ Height,Weight, BMI

☐ Physical Examination

☐ Inclusion Exclusion Criteria

☐ Informed Consent

☐ Collect blood sample

☐ Concomittant medication

☐ Others: __________________

(Omit others if NA)

INVESTIGATOR: ______________________________________

(Inistial & signature)

DATE: ☐☐ ☐☐ ☐☐☐☐ **(dd/mm/yyyy)**
