# Supplementary material for: Optimizing health and nutrition status of migrant construction workers consuming multiple micronutrient fortified rice in Singapore
Source: PLoS One. 2023 Jun 1;18(6):e0285708. doi: 10.1371/journal.pone.0285708 (PMC10234550; doi:10.1371/journal.pone.0285708)
Supplement: S1 File — a English version of health questionnaire. b Tamil version of health questionnaire. c Bengali version of health questionnaire. (ZIP) [file pone.0285708.s005.zip › S4b Tamil version of health questionnaire.pdf]

A. மருத்துவ வரலாறு ஆய்வு

பின்வருவனவற்றில் குறிப்பிடத்தக்க ஒரு வரலாறு உங்களுக்கு இருக்கிறதா?

கெஸ்ட்ரோன்டஸ்டினல் (புண்கள், டிஸ்ஸ்பெசியா)	ஆம்	இல்லை	எனக்கு தெரியாது
கோளாறு உணவு	ஆம்	இல்லை	எனக்கு தெரியாது
சிறுநீரக நோய்	ஆம்	இல்லை	எனக்கு தெரியாது
நாள்பட்ட கல்லீரல் நோய் :	ஆம்	இல்லை	எனக்கு தெரியாது
இருதய நோய் :	ஆம்	இல்லை	எனக்கு தெரியாது
ஹெமாடாலஜி கோளாறு :	ஆம்	இல்லை	எனக்கு தெரியாது
தொற்று நோய் :	ஆம்	இல்லை	எனக்கு தெரியாது
நரம்பியல் :	ஆம்	இல்லை	எனக்கு தெரியாது
சமீபத்திய அறுவை சிகிச்சை (1 வருடத்தில்)	ஆம்	இல்லை	எனக்கு தெரியாது

B. உணவு சப்ளிமெண்ட் & அரிசி நுகர்வு கேள்வித்தாள்

1. கடந்த ஒரு மாதத்தில் எந்த வைட்டமின்கள், தாதுக்கள், மூலிகை பொருட்கள் அல்லது பிற உணவு சப்ளைகளை நீங்கள் உட்கொண்டிருக்கிறீர்களா?

   YES NO

2. நீங்கள் ஒரு வாரம் எத்தனை சாப்பாட்டு நுகர்வோர் அரிசி?

   14 ணவை விட அதிகமாக குறைந்தது 14 உணவுகள்

3. ஒரு வாரத்திற்கு 14 க்கும் மேற்பட்ட உணவை சாப்பிட்டால் மட்டுமே நீங்கள் எந்த உணவு வகை உண்ணுகிறீர்கள்?

   ரொட்டி நூடுல்ஸ் Others: ____________________

C. கல்வி (உயர்ந்த நிலை நிறைவு)

A. உயர்நிலை பள்ளிக்கு குறைவாக
B. உயர்நிலை பள்ளி பட்டதாரி
C. கல்லூரி டிப்ளமோ
