# Supplementary material for: Optimizing health and nutrition status of migrant construction workers consuming multiple micronutrient fortified rice in Singapore
Source: PLoS One. 2023 Jun 1;18(6):e0285708. doi: 10.1371/journal.pone.0285708 (PMC10234550; doi:10.1371/journal.pone.0285708)
Supplement: S1 File — a English version of health questionnaire. b Tamil version of health questionnaire. c Bengali version of health questionnaire. (ZIP) [file pone.0285708.s005.zip › S4c Bengali version of health questionnaire.pdf]

A. চিকিৎসাগত ইতিহাসের সমীক্ষা

আপনার কি নিম্নলিখিতগুলির কোনো উল্লেখযোগ্য ইতিহাস আছে?

গ্যাস্ট্রোইনটেস্টাইনাল (আলসার, ডিস্পেপ্শিয়া)	হ্যাঁ	না	আমি জানি না
খাওয়া সংক্রান্ত অসুস্থতা	হ্যাঁ	না	আমি জানি না
মূত্রাশয়-সম্বন্ধীয় অসুস্থতা	হ্যাঁ	না	আমি জানি না
স্থায়ী যকৃতের ব্যাধি	হ্যাঁ	না	আমি জানি না
কার্ডিওভাসকুলার ব্যাধি	হ্যাঁ	না	আমি জানি না
হেমাটোলজিক অসুস্থতা	হ্যাঁ	না	আমি জানি না
সংক্রামক ব্যাধি	হ্যাঁ	না	আমি জানি না
নিউরলোজিকাল	হ্যাঁ	না	আমি জানি না
সাম্প্রতিক অস্ত্রপোচার (1 বছরের মধ্যে)	হ্যাঁ	না	আমি জানি না

B. খাদ্যের সম্পুরক ও চাল খাওয়া সংক্রান্ত প্রশ্নাবলী

1. আপনি কি কোনো ভিটামিন, খনিজ, ভেষজ পণ্য বা অন্য কোনো খাদ্যের সম্পুরক খেয়েছেন গত এক মাসে?

   হ্যাঁ   না

2. সপ্তাহে কতগুলো খাবারের সাথে আপনি ভাত খান?

   14টির কম খাবার   14টির বেশি খাবার

3. শুধু যদি আপনি সপ্তাহে 14টির কম খাবারে ভাত খান:
   a. কি ধরনের খাবার আপনি খান?

   রুটি   নুডল্স   অন্যান্য: ______________________

C. শিক্ষা (সর্বোচ্চ স্তর সম্পূর্ণ করা)
   a. হাই স্কুলের কম
   b. হাই স্কুলের গ্র্যাজুয়েট
   c. কলেজ ডিপ্লোমা
