# Supplementary material for: Optimizing health and nutrition status of migrant construction workers consuming multiple micronutrient fortified rice in Singapore
Source: PLoS One. 2023 Jun 1;18(6):e0285708. doi: 10.1371/journal.pone.0285708 (PMC10234550; doi:10.1371/journal.pone.0285708)
Supplement: S2 File — (PDF) [file pone.0285708.s006.pdf]

STUDY PROTOCOL

**Study Number**	OBVO-0104DSM
**Title**	**Study to Evaluate the Efficacy of Fortified Rice on Hemoglobin Levels and Levels of Vitamins and Minerals in Healthy Asian Migrant Workers in Singapore**
**Sponsor**	**DSM**
**Country**	Singapore
**Date**	08 Febuary 2018
**Version Number**	**9**

TABLE OF CONTENTS

1. Investigator signature page ... 4
2. Sponsor team signatures page ... 5
3. Study Site ... 6
4. Abbreviations ... 7
5. Synopsis ... 8
6. Study plan ... 11
7. Introduction ... 11
8. Objectives of the trial ... 12
8.1. Primary objective ... 12
8.1.1. Study Intervention ... 12
9. Trial design ... 12
9.1. Type of trial ... 12
9.2. Endpoints ... 13
9.2.1. Study Intervention ... 13
9.3. Subjects, groups and centers ... 13
9.4. Expected study duration ... 13
10. Study Population ... 13
10.1. Description ... 13
10.1.1. Study Intervention: ... 13
10.2. Subject inclusion criteria ... 13
10.2.1. Study Intervention: ... 14
10.3. Subject exclusion criteria ... 14
10.3.1. Study Intervention: ... 14
10.4. Subject withdrawal criteria ... 14
11. Treatment of subjects ... 15
11.1. Study product / Study treatment ... 15
11.2. Concomitant Treatments ... 15
11.3. Subject Compliance ... 16
11.4. Product description ... 16
11.5. Form and dosage ... 16
11.6. Packaging and labeling ... 16
11.7. Product handling ... 17
11.7.1. Storage and distribution ... 17
11.7.2. Product accountability and reconciliation ... 17
11.8. Concomitant diet and treatment ... 17
11.8.1. Permitted concomitant diets / treatments / medications ... 17
11.8.2. Concomitant diets / treatments / medications record ... 17
12. Assessment of Safety ... 18
12.1. Specification of Safety Parameters ... 18
12.2. Intensity ... 19
12.3. Seriousness ... 19
12.4. Relation to study product ... 19
12.5. Unexpected or expected SAE ... 20
12.6. Methods and Timeline of Safety Reporting ... 20
12.7. Procedures for Adverse Event Reporting and Documentation ... 20

12.7.1. Monitoring and Reporting of AE/SAE 20
12.7.2. Follow up..... 20
12.8. Timeline of reporting..... 21
12.9. Reporting 21
13. Conduct of the trial 22
13.1. Subject Enrollment 22
13.2. Study Intervention 23
13.3. Data collection 24
14. Statistics 25
14.1. Sample size calculations 25
14.2. Datasets to be analyzed 25
14.2.1. Intent-to-treat (ITT) analysis dataset..... 25
14.2.2. Per Protocol (PP) analysis dataset..... 25
14.3. Statistical analysis..... 25
15. Legal and ethical prerequisites 26
15.1. Legal requirements 26
15.2. Ethical aspects 26
15.2.1. Protection of the subject's confidentiality 26
15.2.2. Informed consent..... 26
15.2.3. Ethics committee approval 27
15.2.4. Declaration of Helsinki 27
16. Quality control and quality assurance 27
16.1. Monitoring 27
16.2. Quality Control 28
16.2.1. Quality control of essential documents..... 28
16.2.2. Co-monitoring 28
16.3. Audits and inspections 28
16.4. Responsibilities of investigator..... 28
17. Study end procedures 29
17.1. Premature termination of study 29
17.2. Termination of study 29
18. Appendices 29
APPENDIX I 30
APPENDIX II 33
APPENDIX III 34
APPENDIX IV 35
19. References 36

1. Investigator signature page

PRINCIPAL INVESTIGATOR AND CO-INVESTIGATOR

Wei Ying M.D. Scientific
Project Manager SPRIM
Asia Pacific

Ronald Quiambao M.D.
Director for Operations and Clinical Research
SPRIM Philippines

I have read this protocol and agree that it contains all necessary details for carrying out this study. I will conduct the study as outlined herein and will complete the study within the time designated.

I will provide copies of the protocol and all pertinent information to all individuals responsible to me who assist in the conduct of this study. I will discuss this material with them to ensure they are fully informed regarding the study product and the conduct of the study.

I will only use the informed consent form approved by the sponsor or its representative and will fulfill all responsibilities for submitting pertinent information to the Independent Ethics Committee (IEC) responsible for this study.

I agree that the sponsor or its representatives shall have access to any source documents from which case report form information may have been generated.

2. Sponsor team signatures page

Clinical Project Manager

Femke Hannes, PhD

Project Scientific Director

Regina Moench-Pfanner, PhD

Study Site Manager

Kevin Moon

Clinical Data Manager (CRO)

3. Aida Jilani
Digital Innovation Project Manager,
SPRIM Indonesia

3. Study Site

Country / City	International Co-orindation	Institution/CRO
	Dr. Michael Shleifer, PhD	Managing Partner, SPRIM

4. Abbreviations

**ADR**	Adverse Drug Reaction
**AE**	Adverse Event
**ANOVA**	Analysis Of Variance
**ALT**	Alanine Aminotransferase
**AST**	Aspartate Aminotransferase
**BMI**	Body Mass Index
**BP**	Blood Pressure
**CDM**	Clinical Data Manager
**CPM**	Clinical Project Manager
**CRF**	Case Report Form
**CRP**	C Reactive Protein
**CRO**	Contract Research Organization
**EC**	Ethics Committee
**GCP**	Good Clinical Practice
**Hb**	Hemoglobin
**ICH**	International Conference of Harmonization
**IEC**	Independent Ethic Committee
**IRB**	Institutional Review Board
**ITT**	Intent To Treat
**SAE**	Serious Adverse Event
**SOP**	Standard Operating Procedure
**SUSAR**	Serious Unexpected Suspected Adverse Reaction
**TMF**	Trial Master File
**WHO**	World Health Organization
**WMA**	World Medical Association
**WICF**	Written Informed Consent Form

5. Synopsis

**Study Title**	Study to Evaluate the Efficacy of Fortified Rice on Hemoglobin Levels and Levels of Vitamins and Minerals in Healthy Asian Migrant Workers in Singapore
**Study Design**	Single-centre, open label food intervention study
**Study center**	Singapore
**Objective**	**Study Intervention:**<br>• To investigate the change from baseline in the status of hemoglobin (Hb) and key mineral levels including zinc, iron and vitamin B12 and in blood or serum of 180 migrant workers in Singapore following 6 months dietary intervention with fortified rice.<br>• From 180 subjects, 50 subjects will be randomly selected for Homocysteine assessment
**Sample size**	**Study Intervention:**<br>• 180 subjects in a 6-month dietary intervention design, using rice fortified with Fe, Zn, Folate and Vitamins A, B1 (thiamine), B3 (niacin), B6 (pyrodixine) and B12 (cobalmin).<br>• From 180 subjects, 50 subjects will be randomly selected for Homocysteine assessment
**Inclusion and Exclusion criteria**	**<u>Study Intervention:</u>**<br><br>**Inclusion Criteria:**<br>• Healthy adult males between 21 and 50 years old;<br>• Indian/Bangladeshi ethnic origin;<br>• BMI of 17.0 to 27.5 Kg/m2<br>• Currently working in construction site with plans to continue at least the next 06 months<br>• Willing and able to adhere to study procedure and signed an informed consent<br><br>**Exclusion Criteria:**<br><br>• Subject with conditions or disease that investigator consider it is not appropriate to enter the study (e.g. peptic ulcer disease)<br>• Chronic medical illness<br>• History of heavy smoking (more than 10 cigarettes/day continuously for 2 years or more)<br>• Unable to understand Informed consent (translated to Tamil and Bangladesh at basic literacy level)
**Endpoints**	**<u>Study Intervention:</u>**<br><u>Primary endpoint</u>;<br>• Change from baseline in the status of the following:

	a. Whole blood hemoglobin (Hb) status<br>b. Serum ferritin and red Cell folate status<br>c. Serum vitamin B12 status<br>d. Serum zinc Status<br><br><u>Secondary endpoints</u>;<br><br>• Change from baseline in body mass index (BMI)<br>• Homocysteine assessment at endline among those 50 subjects who will be selected at baseline for Homocysteine assessment
**Study Intervention**	**Study Intervention**<br><br>• At baseline (visit 1) - anthropometric measures such as height, weight, BMI and blood pressure, as well as blood samples (10 ml) will be collected prior to dietary intervention.Additional (5ml) of blood sample will be take from those 50 subjects who will be selected randomly for Homocysteine assessment<br>• Informed consent process for the 180 subjects with translators<br>• Subjects (n= 180) will then be provided with cooked fortified rice (45rice) for consumption for 14 meals per week over 26 weeks (approximately 6 months).<br>• By providing fortified rice for only 14 of their weekly meals (lunch and dinner), the subjects will be free to consume 7 meals (breakfast) from other sources provided.<br>• Compliance with the dietary intervention will be monitored on an ongoing basis and collated in a monthly report.<br>• At the end of the intervention, all subjects will undergo the same baseline anthropometric measurements and a blood sample (10 ml) will be collected as performed at baseline.Additional (5ml) of blood sample will be take from those 50 subjects who will be selected randomly for Homocysteine assessment<br>• Subjects will be asked to maintain their habitual lifestyles and refrain from dietary supplements and smoking.

**Visit schedule**	• Visit 1: ICF, Baseline/enrolment; Physical examination, anthropometric measures, blood sample collection.<br>• Visit 2: at 6 months/Endline: Physical examination anthropometric measures, blood sample collection.

6. Study plan

Schedule of Activities	Visit 1	Visit 2
Study Plan	Baseline<br>Enrolment	Endline<br>At 6 months
Information for subjects	X	-
Inclusion/exclusion criteria	X	-
Written consent of subjects	X	-
Subject records and details	X	-
Physical examination (Height, Weight, BMI and Blood Pressure)	X	X
Blood Sample (10 ml)<br>• Additional (5ml) of blood sample will be take from those 50 subjects who will be selected randomly for Homocysteine assessment	X	X
Monitoring report		X
Adverse events and concomitant medications	X	X

7. Introduction

The low nutritional value of meals catered for foreign workers has been the subject of an ongoing study by researchers from the National University of Singapore, academia, civil society leaders and non-governmental organization (NGO). In a survey released in June 2015 (http://www.straitstimes.com/opinion/help-foreign-workers-eat-right), 500 Bangladeshis reported

that they were given unhygienic food to eat and are therefore not eating safely and healthily. Foul-smelling curry, rock-solid fish with scales still intact, and roti prata "so hard that it feels like one is chewing plastic"- are how some foreign workers described the food catered to them at work and construction sites. NGOs working with foreign workers said catered food is a perennial complaint- "The men complained about lack of protein, expired ingredients, and spoiled food". Men arrive in fairly good health and lose weight when they start working- a result of the hard work and long days as well as the poor quality of food.

Construction is a labor-intensive and high-risk activity involving hazardous work. It is physically and mentally demanding, requiring moderate to maximum physical strength and stamina, manual dexterity and coordination, and mental concentration and alertness (Okoro et al, 2016). Manual laborers are some of the hardest working people in the population; poor diet and nutrition can have detrimental effects on their morale, safety, productivity and long-term health according to a report by the International Labour Organization.

Rice fortification is the enrichment of rice with essential vitamins and minerals post-harvesting to increase its nutritional value. Rice is a staple food for more than 3 billion people across the globe. In some countries including Bangladesh, Cambodia and Myanmar, rice contributes as much as 70% of daily energy intake. Rice fortification offers a unique opportunity to substantially improve nutrition and, as such, the health economic status of a large number of people in many countries at a very low cost. Making rice more nutritious through fortification with essential vitamins and minerals is a proven cost-effective intervention to increase micronutrient intake among the general population. (Scaling Up Rice Fortification in Asia; Sight&Life, 2015).

Fortified rice is rice enriched with minerals such as iron, zinc and calcium, and vitamins such as vitamins A, B1, B3, B6, B9, B12, D and E. Fortified rice does not differ from regular rice in terms of appearance, taste or texture.

8. Objectives of the trial

8.1. Primary objective

8.1.1. Study Intervention

The primary objective of this trial is to investigate the change from baseline in the status of hemoglobin (Hb) and some key vitamins and minerals (iron, zinc, folic Acid and vitamin B12) in 180 migrant workers in Singapore following a 6-month dietary intervention with fortified rice.

- Among these 180 subjects, 50 subjects will be randomly selected for Homocysteine assessment

9. Trial design

9.1. Type of trial

This is a single-centre, open label, interventional study on food and nutrition to assess the efficacy of fortified rice on improving the health status of migrant workers.

9.2. Endpoints

9.2.1. Study Intervention

Primary Endpoint:

a. Change from baseline in the status of the following:
- Whole blood hemoglobin (Hb) status
- Serum ferritin and red cell folate status
- Serum vitamin B12 status
- Serum zinc status

Secondary Endpoints:
- Change in BMI
- Homocysteine assessment at endline among those 50 subjects who will be selected at baseline for Homocysteine assessment

9.3. Subjects, groups and centers

Subjects

Study Intervention:

A total of 180 healthy adult males aged between 21 and 50 years old working at a construction site and continuing for the next year (12 months).

Study period

Each subject is enrolled in the study for a period of 6 months.

Centre/s

This is a single-centre study to be conducted in Singapore.

9.4. Expected study duration

The study duration is approximately 6 months or 26 weeks.

10. Study Population

10.1. Description

10.1.1. Study Intervention:

A total of 180 adult males between 21 and 50 years old working at the construction site, who meet the inclusion and exclusion criteria, will participate in the study.

10.2. Subject inclusion criteria

10.2.1. Study Intervention:

All subjects must comply with all the following inclusion criteria:

- Healthy adult males between 21 and 50 years old;
- Indian/Bangladeshi ethnic origin;
- BMI of 17.0 to 27.5 Kg/m2
- Working at the construction site and continuing for one year after enrollment
- Willing and able to adhere to study procedures
- Signed the informed consent form

10.3. Subject exclusion criteria

10.3.1. Study Intervention:

- Subjects with conditions or diseases that investigator considers it is not appropriate to enter the study (e.g. peptic ulcer disease)
- Chronic medical illness
- History of heavy smoking (more than 10 cigarettes/day continuously for 2 years or more)
- Unable to understand Informed conent (translated to Tamil and Bangladesh at basic literacy level)

10.4. Subject withdrawal criteria

Subjects may be withdrawn from the study at any time (i.e. from any further study product or study procedure but not from analysis) for the following circumstances:

1) At their own request.
2) If, in the investigator's opinion, continuation in the study would be detrimental to the subject's wellbeing.
3) Poor compliance, as defined at the discretion of the Investigator (a situation where the subject does not adhere to the study procedures).
4) Protocol deviation(s) which in the opinion of sponsor warrant discontinuation from study; e.g.: violation of inclusion and/or exclusion criteria.

Subjects whose study participation is terminated prematurely for any of the above listed criteria will not be replaced, since a 20% dropout rate is built into the estimated total sample size.

If a subject is withdrawn at any stage during the study due to an adverse event (AE), the supervisor will inform the study site manager who will complete the AE Form and will send it to Clinical Study Manager.

The primary analysis will be a modified intent to treat" m(ITT) analysis; all enrolled subjects who consumed at least one meal of the study product, defined by 600 grams of fortified rice equivalent to 700 grams of cooked fortified rice provided either as lunch or dinner meals will be included in the final efficacy and safety analyses.

The number and percentage of subjects who withdraw early and their reasons for withdrawal will be presented in a table and a CONSORT flow disgram.

The following information will be documented in the source documents and CRF in the event of a subject's withdrawal from the Study, if possible:

i. Date and time of withdrawal
ii. Reasons for withdrawal
iii. Date and time of last consumption of study product
iv. End of study physical examination and blood sample
vi. Follow-up evaluation

If the subject is withdrawn from the study due to an adverse event (AE), and the event has not resolved at the time of withdrawal, additional follow up, as deemed appropriate by the Investigator, will be done and documented in the source document, and the status of the AE at the last follow-up study visit will be captured in the CRF until resolution.

11. Treatment of subjects

11.1. Study product / Study treatment

Fortified rice, refers to the addition of a micronutrients premix* to unfortified rice using any rice fortification technologies, such as hot extrusion, cold extrusion, coating or dusting. For stability reasons and to accommodate local food habits, e.g. washing of rice, hot extrusion is the most suitable technology. (Scaling Up Rice Frotification in Asia, Sight &Life, 2015).

DSM (sponsor) provides the extruded rice kernels (i.e. fortified pellets) to 45Rice which then blends the kernels/ fortified pellets with non- fortified rice at a blending ratio of 1%. The recommended blending ratio of 1% fortified pellets to 99% non-fortified rice grains. For example, 1kg of fortified pellets to be blended with 99kg of rice. After blending, this final rice product made up the IP (investigational product) which would be termed 'study product' for this protocol and study.
The micronutrient premix* is described in Appendix I.

11.2. Concomitant Treatments

Any medication/ treatment taken during the course of the trial will be recorded in the subject CRF, which will include entries for date, type of product taken, frequency, and dosage.

11.3. Subject Compliance

Product compliance will be monitored through: 1.. Assessment of the number of rice meals actually consumed and 2.. indirectly by the supervisors, who is at the study site to provide continuous monitoring on the completion of rice portions during meals.

The study site manager or supervisor conducts compliance monitoring through counting the number of packs of rice/ study product returned by the subject, if any, at each follow-up visit, and during the study.

Compliance is categorized as

1.) Full compliant, having consumed all 14 meals per week;

2.) Moderately compliant, having consumed 8-13 meals per week;

3.) Non-compliant, having consumed less than 8 meals per week. The subject could be withdrawn from study in the event of non-compliance, however, they would still be provided with the study product (free fortified rice).

11.4. Product description

The product used in this trial is fortified rice provided by 45rice. DSM provides the fortified rice kernels and for this study, hot extruded fortified rice kernels will be sponsored. These rice kernels will be blended into fortified rice at a 1% to 99% blending ratio by 45rice who will then distribute the uncooked fortified rice to the the caterer's central kitchen on a weekly or bi-monthly basis. Fortified rice is stored in the premises of the central kitchen's storage area . It is cooked by the caterer on daily basis and delivered to the site for the subjects as cooked fortified rice. Each subject receives 14 weekly meals, namely 7 lunches and 7 dinners for 26-week study period.

11.5. Form and dosage

Subjects will be provided with cooked fortified rice 700 grams each meal for consumption for 14 meals per week over 26 weeks (approximately 6 months).

Other food items that accompany the rice are provided by the caterer from standard menus for two for the ethnic groups, Indian or Bangladeshi. These menus can be found in Appendix III and IV, respectively.

11.6. Packaging and labeling

The rice/ study product is provided as extruded rice kernels by DSM's partner and certified food manufacturer in Thailand. The production of extruded fortified rice kernels takes place in Thailand in batches before it is imported to Singapore. The imported extruded fortified rice kernels are then blended locally in Singapore with unfortified rice at a blending ratio of 1% fortified rice to 99% non-fortified rice (1:99). Blending is done by a third party blender which also packages the rice under the supervision of 45rice, Singapore. The company is licensed under the Singaopore laws on food production per GMP standards.

The final product is then packaged as per natural rice standards in accordance with the AVA's (Agri-food and Vetenary authorithy of Singapore) regulatory guidelines.

A sample of the Package Label is attached in Appendix II. The label contains detailed information on the nutritional composition of the rice and study product.

11.7. Product handling

11.7.1. Storage and distribution

After the study has been approved by the IEC and other regulatory authorities, the study product will be delivered from the supplier's warehouse to the caterer's central kitchen. The study product is stored at the supplier's warehouse in a cool dry place at room temperature and delivered to the central kitchen on a bi-monthly basis.

The central kitchen will prepare the study product by cooking. The rice is cooked and prepared fresh on a daily basis and provided as lunch and dinner. The subjects receive ready packed meals; one pack for each subject containing the fortified rice and other food (see menus Appendices III and IV). An inventory of the study product will be maintained to track the distribution, supply and consumption of the fortified rice and reconciled on a bi-monthly basis.

11.7.2. Product accountability and reconciliation

The Investigator agrees not to supply the study product to any person except the subjects participating in this trial.

Unused study product (uncooked fortified rice) must not be traded or discarded. Unused study product remaining upon completion of the study should be returned to the sponsor.

11.8. Concomitant diet and treatment

11.8.1. Permitted concomitant diets / treatments / medications

It is anticipated that subjects will not be on chronic medication, which could affect metabolism. Otherwise subjects agree to maintain dietary habits throughout the study. It is the investigators' prerogative to assign clinical significance, on a case by case basis.

11.8.2. Concomitant diets / treatments / medications record

Any medication/treatment or food(other than fortified rice) taken during the course of the trial will be recorded in the subject CRF(pages 9 and 16 respectively), which will include entries for date, type of product taken, frequency, and dosage.

12. Assessment of Safety

Safety will be assessed and reported according to ICH-GCP guidelines. All adverse events that occurr during the study will be reported and recorded by study team, whether or not they are considered to be non-serious, serious and/or related to the study product.

12.1. Specification of Safety Parameters

An Adverse Event (AE) is defined as any untoward occurrence in a subject or clinical investigation subject administered a study product and which **does not necessarily** have to have a causal relationship with this treatment.
The AE may be:

- A new illness
- Worsening of a concomitant illness
- An effect of the study product
- A combination of two or more of these factors

Adverse events are illnesses, signs or symptoms (including an abnormal laboratory finding) occurring or worsening in the course of the study. Adverse events can be serious or minor. They may or may not lead to the withdrawal of the subject/ patient from the study. All adverse events must be documented and assessed for intensity, seriousness and relationship to the study product.

All **AEs** occurring during the study will be reported and recorded in the case report form (CRF), regardless of seriousness or relationship to the study product. Investigators must know and record the following information about adverse events:

- Subject and date
- Description of event
- Duration
- Frequency
- Intensity
- Seriousness
- Action taken
- Outcome and sequelae
- Relationship to study product

Surgical procedures themselves are not AEs; they are therapeutic measures for conditions that require surgery. The condition for which the surgery is required is an AE if it occurs or is detected during the study period. Planned surgical measures permitted by the clinical study protocol and the condition(s) leading to these measures are not AEs, if the condition(s) was (were) known before the start of study treatment. In the latter case, the condition should be reported in the medical history.

12.2. Intensity

Mild: Symptoms hardly perceived, only slight impairment of general well-being.

Moderate: Clearly noticeable symptom, but tolerable without immediate relief.

Severe: Overwhelming discomfort.

12.3. Seriousness

The use of the term "adverse event" does not imply a relationship with the study product or with the study. Adverse events fall into the categories "non-serious" and "serious".

A **serious adverse** event is any untoward medical occurrence that at any dose (including overdose):

- Results in death,
- Is life-threatening,
- Requires inpatient hospitalization or prolongation of existing hospitalization,
- Results in persistent or significant disability /incapacity
- Is a congenital anomaly or birth defect

Any other important medical event that may not result in death, be life threatening, or require hospitalization, may be considered a serious AE when, based upon appropriate medical judgment by the principle investigator or the medical doctor in the study team, the event may jeopardize the subject and may require medical or surgical intervention to prevent one of the outcomes listed above.

Non-serious: all other adverse events not corresponding to the definition of serious adverse event are considered as non-serious.

12.4. Relation to study product

The Principal Investigator or the co-Investigator will assess the possibility of a causal relationship between the study product and an adverse event on the basis of the following criteria:

- **Unrelated:** There is an evident other explanation for the AE, e.g.:
  - The AE is obviously explained by the patient's disease;
  - The AE is in accordance with the effect or adverse effect of the concomitant medication;
  - The AE has occurred already prior to the administration of the study product.
- **Unlikely relation:** Reasonable temporal relationship with the intake of the study products, but
  - There is another plausible explanation for the occurrence of the AE.

- **Probable relation:** Reasonable temporal relationship with the intake of the study product and
  - Plausible reasons point to a causal relationship with the study product.
- **Certain relation:** Reasonable temporal relationship with the intake of the study product and
  - There is no other explanation for the AE, and
  - Subsidence or disappearance of the AE on withdrawal of the study product (dechallenge), and
  - Recurrence of the symptoms on rechallenge

12.5. Unexpected or expected SAE

An unexpected AE is an event, the nature or severity of which is not consistent with the applicable product information (e.g.Product Data Sheet, Appendix I) or the assessment of the medical safety officer.

All adverse events that are suspected to be related to the study product and that are both unexpected and serious are considered to be SUSARs (suspected unexpected serious adverse reactions). A SUSAR is to be reported to the regulatory authority within short notice.

12.6. Methods and Timeline of Safety Reporting

The collection of AEs begins when the subjects start consuming the study product and continues throughout the study until last visit. If the investigator detects a serious AE in a study subject after the last visit, and considers the event possibly related to prior study treatment, this should be documented, followed-up and reported.

12.7. Procedures for Adverse Event Reporting and Documentation

Adverse Event /Serious adverse event

12.7.1. Monitoring and Reporting of AE/SAE

Briefing instructions on adverse events monitoring and reporting will be included during the enrollment of the subjects in the prescence of translators. Reporting of adverse event will be done by the subject to foreman who will alert the study site manager. The PI/ physician will be notified accordingly and will evaluate medically and assess to determine their intensity, seriousness and relationship of the adverse events. He/she will record the medical information in the AE/SAE forms in CRF including but not limited to the subject, date, description of event, duration, frequency, intensity, seriousness, action taken, outcome and sequel, relationship to the test product.

12.7.2. Follow up

All AEs/ SAEs must be followed up until the outcome is known.

The Investigator will continue to follow all serious adverse events (SAEs) or other AEs that were regarded as being definitely,probably or possibly related to the study product until they resolve or

stabilise. This follow-up by the Investigator, if required, may extend beyond the end of the study period. All follow-up examinations and/or laboratory findings must be documented and reported.

In the case of serious adverse event(s) persisting beyond trial termination, a follow-up visit may be required. Further, in the event that further analyses are required for the evaluation of a potential cause-effect relationship between the study product and the adverse event, all examinations and laboratory analysis and their results will be documented in the case report forms or in an attached file.

12.8. Timeline of reporting

AE/SAE Procedure/Sponsor: The Study Site Manager must be notified of all adverse events who will then notify the Investigator who would conduct medical evaluation on intensity, seriousness and assign causality or relationship to study product.

AE/SAE procedure/patient: In the event the AE/SAE occur at construction site during off-office hours, the immediate supervisor must bring the patient to the Health Center, 24-hour clinic or A&E (Accident & Emergency department) to seek medical help. The attending physician will arrange referral if necessary to the appropriate hospital specialist for proper management.

AE /SAE reporting timeline

All AEs, if serious or unexpected adverse events, are to be notified within 24 hours. Notification does not depend on whether there is a connection to the study product or not. AE are to be reported with 5 working days of knowledge of the event.

All SAE must be reported to SPRIM by the Investigator within 24 hours of knowledge of the event. SPRIM will notifiy the sponsor DSM within 24 hours of learning the SAE. A final SAE report can follow later.

12.9. Reporting

The sponsor or CRO, on behalf of sponsor should expedite the reporting to all concerned institution(s), to the IRB (S)/IEC(s), where required, and to the regulatory authority (ies) of all adverse drug reactions (ADRs) that are both serious and unexpected.

Such expedited reports should comply with the applicable regulatory requirement(s) and with the ICH Guideline for Clinical Safety Data Management: Definitions and Standards for Expedited Reporting.

The sponsor or CRO , on behalf of the sponsor, should promptly notify all concerned institution(s) and the regulatory authority (ies) of findings that could affect adversely the safety of subjects, impact the conduct of the trial, or alter the IRB/IEC's approval/favorable opinion to continue the trial.

Documentation of a SAE requires that a separate report be completed by the investigator in each case. All SAEs will be reported to the ethics committee, principal investigator, and sponsor.

13. Conduct of the trial

13.1. Subject Enrollment

- The total number of subjects to be enrolled into the fortified rice study is 180. All 180 subjects will receive the study product, which is fortified rice.
- Upon arrival at the Study site, the informed consent process will be conducted. The informed consent process could be facilitated by translators to the subjects on the day of screening.
- A signed written Informed Consent Form (WICF) will be obtained from the subject upon his decision to volunteerily participate in the study.This will be done prior to carrying out any study-related activities, and will be in accordance with all applicable regulatory requirements.
- The Investigator and/or his/her designee will inform the subject, in addition to the WICF, about all aspects of the subject’s study participation, The Independent Ethics Committee (IEC) will approve the WICF. Any amendments to these documents must be approved by the IEC.
- The Investigator and/or his/her designee and the subject must sign and date the WICF prior to any study-related activities being performed. The subject or authorized representative must complete the subject signature with name and date. If an authorized representative signs the WICF, all efforts should be made to obtain an additional electronic signature from the subject himself.
- The decision to participate in the study is entirely voluntary by the subject and/or by the authorized representative. The Investigator and/or his/her designee(s) must emphasize to the subject and/or the authorized representative that consents to participate can be withdrawn at any time, without penalty or loss of benefits to which the subject is otherwise entitled.
- Upon signing the informed consent, the study nurse will conduct initial patient screening. Subject demographics and baseline characteristics and andropoimetric measurements, blood (10ml) will be taken and recorded as the patients’ baseline characteristics. Additional (5ml) of blood sample will be take from those 50 subjects who will be selected randomly for Homocysteine assessment

- The subjects will be directed to the physician for a detailed clinical history and physical examination. The Investigator (PI/co-I) will then determine whether the subject meets all inclusion exclusion criteria for the study before final enrolling the subject.

13.2. Study Intervention

Visit 1: Visit 1: Study intervention:Baseline

1. Informed consent process for 180subjects

2. Collection of demographic information and medical history will be done by the study nurse or healthcare personnel of the study team.

3. Blood samples (10 ml) will be collected from 180 subjects as baseline plasma levels of hemoglobin, Fe, folate, zinc and vitamin B12.

4. Among 180 subjects, 50 subjects will be randomly selected to perform Homocysteine assessment Additional (5ml) of blood sample will be take from those 50 subjects.

5. Subjects who meet the inclusion and exclusion criteria will be eligible to participate in the next steps (points 6 and beyond below) to be enrolled, however subjects who do not meet the screening criteria will not be enrolled.

6. Principal Investigator (PI) or designee with study nurse or designee will conduct physical examination for the subjects enrolled in the study. Anthropometric measures such as height, weight, BMI and blood pressure, as well as blood sampleswill be collected.

7. A unique subject ID number will be assigned to each eligible subject in ascending order. This corresponds to the study subject identification for the fortified rice study and will be used throughout the study conduct. This includes study conduct, safety reporting, compliance with study product and efficacy.

8. Protocol information will then be given by the investigator to potential subjects in the presence of translator who will explain and interpret the English ICF in the following languages: Tamil and Bangladesh.

9. Each subject will sign a consent form that further describes what is entailed by participating in the study, and the need to fully consume the supplied rice that is sponsored (free) at all assigned meals as well as providing subjects with information on how to report any adverse events (AEs) if any.

Monitoring and Compliance

The study is being monitored on an ongoing basis over the six-month period between baseline and endline. The following measures are in place to ensure consistent and regular site-subject contact:

1. Dedicated construction site manager who maintains communication with the subjects and reports any findings to the clinical study manager
2. Constant communication and update to the study manager from process described in point 1 above who would then collate outcomes on monthly basis to the study team
3. Monthly report (such as email) by clinical site manager to PI and study team on well-being, safety, rice consumption, compliance, delivery and logistics
4. Weekly rice inventory report by 45Rice (supplier of fortified rice) which serves as additional layer of rice/ study product compliance tracking
5. Monthly reconciliation in CRF by clinical data manager on rice meals full consumption.

This communication and monitoring with the subjects is ongoing between the construction site manager and study manager team.

Visit 2: At the end of 6 months/Endline

1. Anthropometric measures such as height, weight, BMI and blood pressure, as well as blood samples (10 ml) will be collected. Additional (5ml) of blood sample will be take from those 50 subjects who will be selected randomly for Homocysteine assessment

2. The blood biochemistry assesses levels of Hb, Fe, folate, Zn and B12 after 6 months.
3. The clinical data manager will collate the monitoring reports.
4. Adverse events reporting and reconciliation.
5. The PI conducts medical examination with anthropometric measurements.

13.3. Data collection

Paper CRFs will be provided for each patient, to be filled out by the Investigator or designated personnel. CRFs will be signed by the investigator

when they are complete.

14. Statistics

14.1. Sample size calculations

A sample size of 180 subjects is estimated under the following considerations:

- Two-sided alpha: 5% (overall)
- Maximum anticipated subject attrition: 20%
- Effect size: at least 0.30 for all parameters

14.2. Datasets to be analyzed

14.2.1. Intent-to-treat (ITT) analysis dataset

All subjects who receive at least one dose of study product will be included in the efficacy and safety analyses, in accordance with the modified Intent-to-treat (ITT) principle.

14.2.2. Per Protocol (PP) analysis dataset

All subjects who have no protocol violations (protocol deviations are permitted) and remained on study for the full product intervention period of 26 weeks, and were fully or moderately compliant with intake of study product will be included for PP analysis. Compliance with study product (fortified rice) is defined as full compliance, moderate compliance or non compliance based on the number of meals of fortified rice/ study product consumed:
Non compliance (<8 meals week)= (<208 meals over 26 weeks)
Moderately compliance (8-13 meals per week) =(208-338 meals over 26 weeks)
Full compliance (14 meals per week)= (364 meals over 26 weeks)

Only efficacy analysis will be performed in this population.

Protocol violations and deviations, study product consumption and study completion data will be evaluated during the data review to define the PP dataset before database lock.

14.3. Statistical analysis

Descriptive statistics will be provided for demographics and baseline characteristics. Baseline levels of serum hemoglobin (Hb), iron, zinc, folic acid and vitamin B12 will be presented as means, medians, standard deviations, minima and maxima and further characterized as “low’ (below the commonly accepted lower limit of normal) or “normal” and the baseline number and percent in each category reported.

Changes in blood/serum levels of hemoglobin, iron, zinc,folic acid, vitamin B12 and Homocystein from 50 randomly selected subjects will be analysed.Percent of subjects being nutrient deficient will be calculated by subtracting the baseline value from the end-of-the-

study value. Change in each variable will be analyzed using the paired t-test or Wilcoxon test, as appropriate to the data distributions. The data for each laboratory parameter will also be analyzed using the McNemar's test to examine the shift from "low" to "normal" levels during the study period.

Subgroups of ethnicity, age, and BMI may also be analyzed in the same manner.

Subject compliance with study product, as defined in Section 14.2.2 will be calculated for each subject and the number and percent of subjects in each compliance category will be presented descriptively. Adverse events will be classified by preferred term and body system using MedDRA, and tabulated by severity and relationship. Serious adverse events will be discussed in narratives. Concomitant and pre-study medications will be listed by subject ID, summarized by medication class and treatment group, and listed by treatment group.

15. Legal and ethical prerequisites

15.1. Legal requirements

The study will be conducted after approval from local Singapore IRB and AVA (Agri-Food & Veterinary Authority of Singapore).

15.2. Ethical aspects

15.2.1. Protection of the subject's confidentiality

Confidentiality of all study participants will be maintained; codes for subject identification will be utilized.

15.2.2. Informed consent

Signed Written Informed Consent by the subject upon confirming his eligibility for the study is required. This will be done prior to carrying out any study-related activities and will be done in accordance with all applicable regulatory requirements.

The Investigator and/or designee will inform the subject in addition to the WICF about all aspects of the subject's study participation. The Independent Ethics Committee (IEC) will approve the WICF. Any amendments to these documents must be approved by the IEC.

The Investigator and/or his/her designee and the subject and/or the subject's legal authorized representative (next of kin, other authorized individual) must sign and date the WICF prior to any study related activities are performed. The subject or the authorized representative must complete the printed name and enter the date of signature him or herself. If an authorized representative signs the WICF, all efforts should be made to obtain an additional signature from the subject him or herself.

The decision to participate in the study is entirely voluntary by the subject. The investigator and/or his/her designee must emphasize to the subject and/or the authorized

representative that the consent to participate can be withdrawn at any time without penalty or loss of benefits to which the subject is otherwise entitled.

15.2.3. Ethics committee approval

The study will be conducted after approval from the Parkway Independent Ethic Committee and AVA (Agri-Food & Veterinary Authority of Singapore). Commencement of the clinical trial is not permitted without written approval of the ethics committee.

The IRB/IEC must be notified of all subsequent additions or changes in the study protocol. Notification of the IRB / IEC is also required in the event of a SAE during the clinical trial.

15.2.4. Declaration of Helsinki

This trial will be conducted according to the principles and rules laid down in the Declaration of Helsinki and its subsequent amendments.

16. Quality control and quality assurance

16.1. Monitoring

Regular monitoring visits by representatives of the sponsor (45rice) will be made during the study and the monitoring activities will be detailed in a monitoring plan.

Monitoring will begin with an initiation visit prior to study commencement to clarify all aspects of the protocol and documentation. The purpose of later visits during the implementation period will be to evaluate study progress, adherence to study protocol and to Good Clinical Practice (GCP) guidelines and regulation.

Protocol deviations will be reported in the monitoring report and eventual corrective action plan implemented. The main protocol deviations that will be looked for are:

- Informed consent process not adequately performed;
- Violation of Inclusion Exclusion criteria;
- Non-compliance with study product storage, dispensation, allocation, use, or return requirements;
- Intake of unauthorized concomitant diets/treatments/medications;
- Study visit schedule not followed;
- Any other GCP non-compliance

The monitor will communicate any detected protocol deviations to the investigator, who must fill in a protocol deviation form. The signed form will be sent by e-mail to the Clinical Project Manager and study team. According to IEC requirements, the CRA or clinical data manager should ensure that the site or investigator will properly notify the IEC.

At the end of the trial the CRA or Clinical data manager will make a study closing visit to all sites to ensure that all documentation is complete and that the trial master file (TMF) is complete. In all cases, it is the responsibility of the CPM, Clinical data manager and study team to maintain subject confidentiality.

16.2. Quality Control

16.2.1. Quality control of essential documents

The sponsor or designee will implement and maintain quality assurance and quality control systems with Standard Operating Procedures (SOPs) to ensure that this clinical trial is conducted and data are generated, documented (recorded) and reported in compliance to the protocol, Good Clinical Practice (GCP) standards, ICH and other applicable local regulations.

16.2.2. Co-monitoring

Co-monitoring visits might be performed by the sponor with the monitor from CRO as deemed necessary.

16.3. Audits and inspections

Inspection

An inspection is defined as the act of a regulatory authority of conducting an official review of documents, facilities, records and any other resources that are deemed by the authorities to be related to the clinical trial and that may be located at the site of the trial, or at the sponsors and or CRO facilities or any other establishments deemed appropriate by the regulatory authorities.

Audit

An audit is a systematic and independent review of trial-related activities and documents to determine whether trial-related activities were conducted and the data were accurately recorded and analyzed according to the protocol, SOPs, GCP and appropriate requirements. In conducting this study, the Investigator accepts that the sponsor, ethical committee or regulatory body may at any time by appointment conduct an audit of the study site.

16.4. Responsibilities of investigator

The investigators are responsible for the following:

- Obtaining the written and dated approval of the applicable ethics committee (and other regulatory agency, if any) prior to the conduct of the study.
- Selection of participants/ subjects in accordance with the inclusion and exclusion criteria; and after obtaining written informed consent of the subject.

- Maintain confidentiality and safety of subjects in accordance with the Declaration of Helsinki.
- Adherence to the study protocol and the spirit of GCP. If modification becomes necessary, the rationale will be provided in a protocol amendment signed by the investigator and sponsor for submission to the ethics committee.
- Accurate, complete and timely data reported to the sponsor via CRF.
- During the course of the trial, provide subjects with any newly available information that may be relevant to them.
- Identification of serious adverse events with notification to sponsor, ethics committee and health authorities, as applicable.
- Co-operation with monitoring visits, audits and regulatory inspections. Providing direct access to source data and documents.

17. Study end procedures

17.1. Premature termination of study

Should it prove necessary to discontinue the study permanently prior to completion, the sponsors will notify the investigators and additional contacts, including the IRB/IEC, of the rationale. All relevant study documents will then be returned to the sponsor, and the study product will be destroyed or returned.

17.2. Termination of study

After the completion or termination of the study, the Investigator will inform the IEC of the end of the study. A certificate of study closure will be issued. Every serious or unexpected adverse event that might affect a subject’s safety must be brought to the IEC attention, as required by the IEC regulations. In addition, every report concerning an adverse event that the Investigator sent to the sponsor must be sent by the latter to the relevant IEC.

18. Appendices

APPENDIX I

DSM Nutritional Products Asia Pacific 30 Pasir Panjang Road #13-31, Singapore 117440 Tel: (+65) 6632 6500 Fax: (+65) 6632 6600 www.dsmnutritionalproducts.com

Product Data Sheet

Product name Product code

Description

Vitamin Mineral Premix 45R-PP 5041311001

This product consists of off-white to pale yellow free-flowing pellets that contain high concentration of vitamins and minerals. These pellets are intended for reconstitution into fortified rice by blending with conventional rice.

Composition

Rice Flour (> 90%), Vitamin Mineral Premix*

* Vitamin Mineral Premix contains the following ingredients in descending order of quantity: Ferric Pyrophosphate, Niacinamide, Zinc Oxide, Monoglyceride, Carrier (Maltodextrin), Vitamin A Palmitate, Pyridoxine Hydrochloride, Thiamine Mononitrate, Folic Acid, Vitamin B12 (cyanocobalamin).

Specifications for fortified pellets

Appearance: Moisture: Length Width: Thickness: Vitamin A: Vitamin B1: Vitamin B6: Vitamin B12: Folic Acid: Niacinamide: Iron:

Zinc: Microbiological: Aerobic count Yeasts and moulds Coliform

Px-Ref: XW80271000

Free-flowing pellets Max. 14.5% 3.9-5.3mm 1.4-2.6mm 1.2-2.6mm

Min. 40,000 IU / 100g fortified pellets Min. 40 mg / 100g fortified pellets Min. 48 mg / 100g fortified pellets Min. 80 µg / 100g fortified pellets Min. 10,400 µg / 100g fortified pellets Min. 560 mg / 100g fortified pellets Min. 320 mg / 100g fortified pellets Min. 480 mg / 100g fortified pellets

Max. 1000 cfu/g Max. 100 cfu/g Negative in 1 g

Apr 26, 2017 Revision 00

DSM Nutritional Products Asia Pacific 30 Pasir Panjang Road #13-31, Singapore 117440 Tel: (+65) 6632 6500 Fax: (+65) 6632 6600 www.dsmnutritionalproducts.com

Product Data Sheet

Escherichia coli Staphylococcus aureus Heavy metals: Arsenic Lead Mercury

Nutritional Information Nutrient

Energy Protein Total Fat Carbohydrate Fibre

Sodium Vitamin Vitamin Vitamin Vitamin Vitamin Vitamin Iron Zinc

Negative in 1 g Negative in 0.1 g

Max. 1 mg/kg Max. 1 mg/kg Max. 0.2 mg/kg

Typical Values Per 100g fortified pellets

340 kcal 5.7 g 1.9 g

83.2 g 0.5 g 7.3 mg

A 50,000 IU B1 50 mg B6 60 mg B12 100 µg B9 13,000 µg B3 700 Mg

400 Mg

600 Mg Note: Nutritional information values stated above are derived based on theoretical calculation.

Uses

For rice fortification.

Recommended blending ratio of fortified pellets in fortified rice is 1%, for example 1 kg fortified pellets to be blended with 99 kg rice.

DSM Nutritional Products Asia Pacific 30 Pasir Panjang Road #13-31, Singapore 117440 Tel: (+65) 6632 6500 Fax: (+65) 6632 6600 www.dsmnutritionalproducts.com

Product Data Sheet

Stability and storage

This product may be stored for 12 months from the date of manufacture in its unopened, original container at a temperature below 25°C.

The product should be stored under dry, clean condition and away from heat, oil, odorous and toxic materials. The area must have pest control program in place to prevent rodent and insect infestation.

Packaging

Packed in 25kg compound craft bag lined with PE bag.

Safety

This product is safe for the intended use. Read and understand product data sheet before using this product.

Legal notice

All information given in this product data sheet is based on our current knowledge and experience, and correct at the time of issue. However, it is subjected to seasonal variation and revision. Information provided is typical data only. It does not relieve you from carrying out your own precautions and tests. We do not assume any liability in connection with your product or its use. You must comply with all applicable laws and regulation, and observe all third part rights.

APPENDIX II

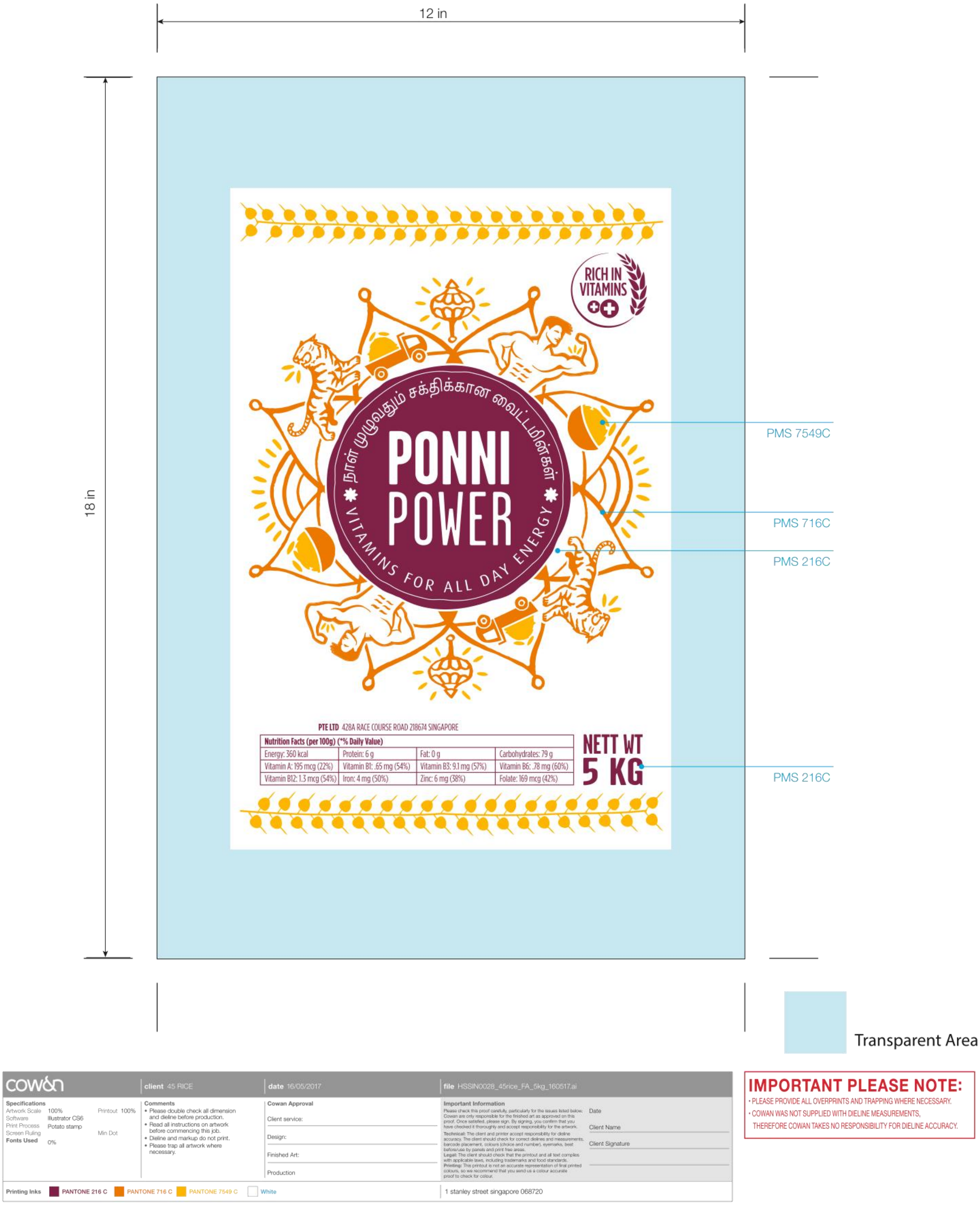
12 in
18 in
RICH IN VITAMINS
நாள் முழுவதும் சக்திக்கான வைட்டமின்கள்
PONNI POWER
VITAMINS FOR ALL DAY ENERGY
PMS 7549C
PMS 716C
PMS 216C
PTE LTD 428A RACE COURSE ROAD 218674 SINGAPORE
Nutrition Facts (per 100g) (*% Daily Value)
Energy: 360 kcal | Protein: 6 g | Fat: 0 g | Carbohydrates: 79 g
Vitamin A: 195 mcg (22%) | Vitamin B1: .65 mg (54%) | Vitamin B3: 9.1 mg (57%) | Vitamin B6: .78 mg (60%)
Vitamin B12: 1.3 mcg (54%) | Iron: 4 mg (50%) | Zinc: 6 mg (38%) | Folate: 169 mcg (42%)
NETT WT 5 KG
PMS 216C
Transparent Area
cowan
client 45 RICE
date 16/05/2017
file HSSIN0028_45rice_FA_5kg_160517.ai
Specifications
Artwork Scale 100% Printout 100%
Software Illustrator CS6
Print Process Potato stamp
Screen Ruling Min Dot
Fonts Used 0%
Comments
• Please double check all dimension and dieline before production.
• Read all instructions on artwork before commencing this job.
• Dieline and markup do not print.
• Please trap all artwork where necessary.
Cowan Approval
Client service:
Design:
Finished Art:
Production
Important Information
Please check this proof carefully, particularly for the issues listed below. Cowan are only responsible for the finished art as approved on this proof. Once satisfied, please sign. By signing, you confirm that you have checked it thoroughly and accept responsibility for the artwork.
Technical: The client and printer accept responsibility for dieline accuracy. The client should check for correct dielines and measurements, barcode placement, colours (choice and number), eyemarks, best before/use by panels and print free areas.
Legal: The client should check that the printout and all text complies with applicable laws, including trademarks and food standards.
Printing: This printout is not an accurate representation of final printed colours, so we recommend that you send us a colour accurate proof to check for colour.
Date
Client Name
Client Signature
Printing Inks PANTONE 216 C PANTONE 716 C PANTONE 7549 C White
1 stanley street singapore 068720
IMPORTANT PLEASE NOTE:
• PLEASE PROVIDE ALL OVERPRINTS AND TRAPPING WHERE NECESSARY.
• COWAN WAS NOT SUPPLIED WITH DIELINE MEASUREMENTS, THEREFORE COWAN TAKES NO RESPONSIBILITY FOR DIELINE ACCURACY.

APPENDIX III

Proposed Menu For Indian Food			
Sunday to Saturday			
DAY	BreakFast	Lunch	Di nner
Sunday Non Veg	Chappa ti Kurma	Nandu Curry Muttai Samba! Rice+Butter Milk	Karuvattu Curry Chan na Masai Rice+Rasam
Sunday Veg	Chappati Kurma	Vegetable Ku nua +Channa Masala +Rice+Butter Milk	Sambar + Moormelagai Veg Masala Paratal Spicy Rice + Rsam
Monday Non Veg	Roti prata + **Kuruma**	Fish Curry + Fish Fry + Rice +Butter Milk	Chicken Curry + Veg Rice + Rasam
Monday Veg	Roti prata + Ku ruma	VathalCurry+PotatoFry**/** Biter Gourd+ Rice + Butter Milk	Sambar+Veg Moormelagai **/** Appalam Rice Rasam
Tuesday Veg	Pulli Rice	Pulli Curry Cabbage **/** Saraka Saraka **/** Cabbage Kottu Rice+Butter Milk	Sambar + Rava Palpayasam Potato Green Peas Paratal Rice + Rasam
Wednesday Non Veg	Chappati Kurma	Fish Cu rry + Fish Fry +Rice +Butter Milk	Pepper Chicken Chicken Dalcha Rice+Rasam
Wednesday Veg	Chappati Kurma	Moor Curry Ca uliflower Fry Moormelagai Rice+Butter Milk	Sambar + Cabbage Kuttu Moormelagai **/** Appalam + Rice Rasam
Thursday Non Veg	Ediyappam Sugar	Chicken Fry + Sambar + Rice+Butter Milk	Mutton Curry + Mutton Masala+Veg+Rice+Rasam
Thursday Veg	Ediyappam Sugar	Samba r +Moormelagai Pakoda **/** Bitter Gourd fry Rice + Butter Milk	Dhall Thaduka + Califlower + Potato Masala Paratal Rice + Rasam
Friday	Lemon Rice	Moor Curry Pnli Manthi(brinjal,Vendi, Pottato,Mochai) ParatalMoormelagai **/** AppalamRice+ Rasam	Sambar+Potato Masala Paratal Kesa ri **/** Pa ruppn Payasam **/** YellowRavapaysam**/**Rice+ Rasam
Saturday Non Veg	Dhosai **/** Idly + Dhall	Fish Curry + Fish Fry Rice+Butter Milk	Briyaui Rice Chicken Cu rry/K uru ma Raitha Salad (Cumcumber,Oninon, Yogurt)
Saturday Veg	Dhosai **/** Id ly + Dhall	Puli Cu rry + Brinjal Fry + Rice +Butter Milk	Veg Briyani Vegetable Kurma + Brinjal Fry+ Raitha Salad (Cumcumber, Onion, Carrot, Y ogurt)

APPENDIX IV

Proposed Menu For Bangala Food<br>Monday to Sunday				
DAY	Break Fast	Lunch	Dinner	Remarks
Monday	Parta/Chapthi x 02 pcs<br>Chana dhal or Sugi or Bhaji	Masoor Dhal<br>Fish curry With Vegetables<br>Rice	Masoor Dhal<br>Bhaji or Botah<br>Mutton curry<br>Rice	Mutton curry can be replaced by Chicken Curry or Egg curry or Fish Curry due to religious or other reasons
Tuesday	Parta/Chapthi x 02 pcs<br>Chana dhal or Sugi or Bhaji	Masoor Dhal<br>Fish curry With Vegetables<br>Rice	Masoor Dhal<br>Bhaji or Botah<br>Beef curry<br>Rice	Beef curry can be replaced by Chicken Curry or Egg curry or Fish Curry due to religious or other reasons
Wednesday	Parta/Chapthi x 02 pcs<br>Chana dhal or Sugi or Bhaji	Masoor Dhal<br>Fish curry With Vegetables<br>Rice	Masoor Dhal<br>Bhaji or Botah<br>Chicken curry<br>Rice	
Thursday	Parta/Chapthi x 02 pcs<br>Chana dhal or Sugi or Bhaji	Masoor Dhal<br>Fish curry With Vegetables<br>Rice	Masoor Dhal<br>Bhaji or Botah<br>Beef curry<br>Rice	Beef curry can be replaced by Chicken Curry or Egg curry or Fish Curry due to religious or other reasons
Friday	Parta/Chapthi x 02 pcs<br>Chana dhal or Sugi or Bhaji	Masoor Dhal<br>Fish curry With Vegetables<br>Rice	Masoor Dhal<br>Bhaji or Botah<br>Mutton curry<br>Rice	Mutton curry can be replaced by Chicken Curry or Egg curry or Fish Curry due to religious or other reasons
Saturday	Parta/Chapthi x 02 pcs<br>Chana dhal or Sugi or Bhaji	Masoor Dhal<br>Fish curry With Vegetables<br>Rice	Masoor Dhal<br>Bhaji or Botah<br>Chickencurry<br>Rice	
Sunday	Parta/Chapthi x 02 pcs<br>Chana dhal or Sugi or Bhaji	Masoor Dhal<br>Fish curry With Vegetables<br>Rice	Masoor Dhal<br>Bhaji or Botah<br>Beef curry<br>Rice	Beef curry can be replaced by Chicken Curry or Egg curry or Fish Curry due to religious or other reasons

Note :
The breakfast menu will be differ upon the availability
To the best we will follow the above menu but depends upon the availability and stock of the ingredient.
There may be some changes in the above menu then and there

19. References

1. Moretti, D., Zimmerman, M.B. Muthayya, S., et.al., 2006. Extruded Rice Fortified with Micronized Ground Ferric Pyrophosphate Reduces Iron Deficiency in Indian Schoolchildren: A Double-blind Randomized Controlled Trial. *American Journal of Clinical Nutrition,* Vol. 84 no. 4, 822-829. Available at: http://ajcn.nutrition.org/content/84/4/822.long

2. Radhika, M.S., Nair, K.M., et.al., 2011. Micronized Ferric Pyrophosphate Supplied Through Extruded Rice Kernels Improves Body Iron Stores in Children: A Double-blind, Randomized, Placebo-controlled Midday Meal Feeding Trial in Indian Schoolchildren. *American Journal of Clinical Nutrition,* Vol. 94 no.5, 1202-1210. Available at: http://ajcn.nutrition.org/content/94/5/1202.long

3. Hackl, L., Cercamondi, C.I., et.al., 2016. Cofortification of Ferric Pyrophosphate and Citric Acid/Trisodium Citrate into Extruded Rice Grains Doubles Iron Bioavailability Through In Situ Generation of Soluble Ferric Pyrophosphate Citrate Complexes. *American Journal of Clinical Nutrition,* 115.128173. Available at: http://ajcn.nutrition.org/content/early/2016/04/06/ajcn.115.128173.full.pdf+html

4. Agdeppa, I.A., Capanzana, M.V., et. Al., 2008. Efficacy of Iron-Fortified Rice in Reducing Anemia Among Schoolchildren in the Philippines. *International Journal for Vitamin and Nutrition Research,* 78, pp.74-86. Available at: http://econtent.hogrefe.com/doi/pdf/10.1024/0300-9831.78.2.74

5. Chavasit, V., Porasuphatana S., et. Al., 2015. Iron Bioavailability in 8-24-Month-Old Thai Children From A Micronutrient-Fortified Quick-Cooking Rice Containing Ferric Ammonium Citrate or A Mixture of Ferrous Sulphate and Ferric Sodium Ethylenediaminetetraacetic Acid. *Maternal and Child Nutrition,* 11 (Suppl.4), pp. 179-187. Available at: http://onlinelibrary.wiley.com/doi/10.1111/mcn.12167/full

6. Food Fortification Initiative (FFI) Efficacy and Effectiveness studies. Rice fortification's impact on nutrition. Atlanta, USA: FFI, 2014. Available at: http://www.ffinetwork.org/about/faq/documents/Rice_Fortification_Nutrition_ImpactOct2014.pdf [Accessed 18 July, 2017].

7. Hotz, C., Porcayo, M., et.al., 2008. Efficacy of Iron-Fortified Ultra Rice in Improving the Iron Status of Women in Mexico. *Food and Nutrition Bulletion*, Vol. 29, no. 2. Available at: http://journals.sagepub.com/doi/abs/10.1177/156482650802900208?url_ver=Z39.88-2003&rfr_id=ori:rid:crossref.org&rfr_dat=cr_pub%3dpubmed

8. Perignon, M., Fiorentino, M., et.al, 2016. Impact of Multi-Micronutrient Fortified Rice on Hemoglobin, Iron and Vitamin A Status of Cambodian Schoolchildren: A Double-Blind Cluster-Randomized Controlled Trial. *Nutrients,* 8 (1) :29. Available at: https://www.ncbi.nlm.nih.gov/pmc/articles/PMC4728643/

9. Beinner, M., Velasquez-Melendez, G., et.al, 2009. Iron-Fortified Rice as Efficacious as Supplemental Iron Drops in Infants and Young Children. *The Journal of Nutrition.* Available at: http://jn.nutrition.org/content/early/2009/11/04/jn.109.112623

10. Thankachan, P., Hyun Rah, J., et.al, 2012. Multiple Micronutrient-Fortified Rice Affects Physical Performance and Plasma Vitamin B-12 and Homocysteine Concentrations of Indian School Children. *The Journal of Nutrition,* vol.142 no. 5, pp.846-852. Available at: http://jn.nutrition.org/content/142/5/846.full

11. Pinkaew, S., Winichagoon, P., et.al, 2013. Extruded Rice Grains Fortified with Zinc, Iron, and Vitamin A Increase Zinc Status of Thai School Children When Incorporated into a School Lunch Program. *The Journal of Nutrition,* vol. 143, no. 3, pp. 362-368. Available at: http://jn.nutrition.org/content/143/3/362

12. Okoro, C., Musonda, I., & Agumba, J., 2016. Identifying Factors Influencing Construction Workers' Food Choices in Gauteng, South Africa: A Pilot Investigation. *University of Johannesburg.* Available at: https://ujcontent.uj.ac.za/vital/access/manager/Repository/uj:20639 [Accessed 05 April, 2017].
