# Supplementary material for: Optimizing health and nutrition status of migrant construction workers consuming multiple micronutrient fortified rice in Singapore
Source: PLoS One. 2023 Jun 1;18(6):e0285708. doi: 10.1371/journal.pone.0285708 (PMC10234550; doi:10.1371/journal.pone.0285708)
Supplement: Attachment — Submitted filename: Response to Reviewers.docx [file pone.0285708.s007.docx]

REBUTTAL LETTER

Dear Editor and Reviewers,

**We have addressed all your comments and have inserted our responses in italics into the comments; we have accordingly edited the manuscript. We have also provided the amended statements and other information as per below requirements by the editor in our cover letter.**

Kind regards,

Regina Moench Pfanner

PONE-D-21-12903

Optimizing health and nutrition status of migrant construction workers consuming multiple micronutrient fortified rice in Singapore

PLOS ONE

Dear Dr. Moench Pfanner,

Thank you for submitting your manuscript to PLOS ONE. After careful consideration, we feel that it has merit but does not fully meet PLOS ONE’s publication criteria as it currently stands. Therefore, we invite you to submit a revised version of the manuscript that addresses the points raised during the review process.

The manuscript has been evaluated by two reviewers, and their comments are available below.

The reviewers have raised a number of major concerns. They request copyediting to ensure the language and text of the study is more readable and easy to follow. The reviewers also note concerns about the statistical analyses presented, and they request more appropriate tests to be used and a re-analyses of the data to be completed. *We have addressed these comments and answered below to these comments.*

Could you please carefully revise the manuscript to address all comments raised?

Please submit your revised manuscript by Apr 03 2022 11:59PM. If you will need more time than this to complete your revisions, please reply to this message or contact the journal office at [plosone@plos.org](mailto:plosone@plos.org). When you're ready to submit your revision, log on to <https://www.editorialmanager.com/pone/> and select the 'Submissions Needing Revision' folder to locate your manuscript file.

Please include the following items when submitting your revised manuscript:

- A rebuttal letter that responds to each point raised by the academic editor and reviewer(s). You should upload this letter as a separate file labeled 'Response to Reviewers'.
- A marked-up copy of your manuscript that highlights changes made to the original version. You should upload this as a separate file labeled 'Revised Manuscript with Track Changes'.
- An unmarked version of your revised paper without tracked changes. You should upload this as a separate file labeled 'Manuscript'.

If you would like to make changes to your financial disclosure, please include your updated statement in your cover letter. Guidelines for resubmitting your figure files are available below the reviewer comments at the end of this letter.

If applicable, we recommend that you deposit your laboratory protocols in protocols.io to enhance the reproducibility of your results. Protocols.io assigns your protocol its own identifier (DOI) so that it can be cited independently in the future. For instructions see: <https://journals.plos.org/plosone/s/submission-guidelines#loc-laboratory-protocols>. Additionally, PLOS ONE offers an option for publishing peer-reviewed Lab Protocol articles, which describe protocols hosted on protocols.io. Read more information on sharing protocols at <https://plos.org/protocols?utm_medium=editorial-email&utm_source=authorletters&utm_campaign=protocols>.

We look forward to receiving your revised manuscript.

Kind regards,

Jamie Royle
Associate Editor
PLOS ONE

Journal Requirements:

When submitting your revision, we need you to address these additional requirements.

1. Please ensure that your manuscript meets PLOS ONE's style requirements, including those for file naming. The PLOS ONE style templates can be found at 
<https://journals.plos.org/plosone/s/file?id=wjVg/PLOSOne_formatting_sample_main_body.pdf> and 
<https://journals.plos.org/plosone/s/file?id=ba62/PLOSOne_formatting_sample_title_authors_affiliations.pdf>

*This has been done and we edited the Tables 1-3 into PLOS ONE’s style requirement.*

2. Thank you for submitting your clinical trial to PLOS ONE and for providing the name of the registry and the registration number. The information in the registry entry suggests that your trial was registered after patient recruitment began. PLOS ONE strongly encourages authors to register all trials before recruiting the first participant in a study.

As per the journal’s editorial policy, please include in the Methods section of your paper:
a) your reasons for your delay in registering this study (after enrolment of participants started); *The trial was registered after subject recruitment began due to a professional oversight; the authors confirm that all ongoing and related trials for this drug/intervention are registered. Manuscript was edited accordingly.*
b) confirmation that all related trials are registered by stating: “The authors confirm that all ongoing and related trials for this drug/intervention are registered". *This was done and manuscript edited accordingly.*

3. Please include additional information regarding the survey or questionnaire used in the study and ensure that you have provided sufficient details that others could replicate the analyses. For instance, if you developed a questionnaire as part of this study and it is not under a copyright more restrictive than CC-BY, please include a copy, in both the original language and English, as Supporting Information. If the original language is written in non-Latin characters, for example Amharic, Chinese, or Korean, please use a file format that ensures these characters are visible. *The health questionnaire that was used at the start of the intervention has been up-loaded as supporting information in English, Tamil and Bengali. File names: S4a English version of health questionnaire, 4b Tamil version of health questionnaire, 4c Bengali version of health questionnaire.*

4. Please state whether you validated the questionnaire prior to testing on study participants. Please provide details regarding the validation group within the methods section. *We inserted the following sentence into the manuscript: We were not able to validate the health questionnaire as the migrant workers were not available due to their working schedule for any additional time except for the health assessment at the start of the intervention.*

5. Please expand the acronym “DSM” (as indicated in your financial disclosure) so that it states the name of your funders in full. *We have provided this information in the cover letter.*
This information should be included in your cover letter; we will change the online submission form on your behalf.

6. Thank you for stating the following in the Competing Interests section: "The authors have declared that no competing interests exists for this publication. However, two authors have been supported financially in the beginning of the study by either being an employee of DSM (FH) or an independent consulting firm (RMP) to give input to the study design. At the time of the study implementation one author (KM) was working for 45RICE, a social enterprise dedicated to improving lives through nutrition. DSM’s financial support had no influence on the statistical analysis which was conducted independently and on writing of the article. The authors alone are responsible for the views expressed in this publication."

We note that one or more of the authors have an affiliation to the commercial funders of this research study: Ibn360 and 45RICE

a. Please provide an amended Funding Statement declaring this commercial affiliation, as well as a statement regarding the Role of Funders in your study. If the funding organization did not play a role in the study design, data collection and analysis, decision to publish, or preparation of the manuscript and only provided financial support in the form of authors' salaries and/or research materials, please review your statements relating to the author contributions, and ensure you have specifically and accurately indicated the role(s) that these authors had in your study. You can update author roles in the Author Contributions section of the online submission form. *An amended Funding Statement is in the cover letter.*

Please also include the following statement within your amended Funding Statement. 
“The funder provided support in the form of salaries for authors [insert relevant initials], but did not have any additional role in the study design, data collection and analysis, decision to publish, or preparation of the manuscript. The specific roles of these authors are articulated in the ‘author contributions’ section.”

If your commercial affiliation did play a role in your study, please state and explain this role within your updated Funding Statement.

b. Please also provide an updated Competing Interests Statement declaring this commercial affiliation along with any other relevant declarations relating to employment, consultancy, patents, products in development, or marketed products, etc.  *The amended competing interest Statement is in the cover letter.*

Within your Competing Interests Statement, please confirm that this commercial affiliation does not alter your adherence to all PLOS ONE policies on sharing data and materials by including the following statement: "This does not alter our adherence to  PLOS ONE policies on sharing data and materials.” (as detailed online in our guide for authors <http://journals.plos.org/plosone/s/competing-interests>). If this adherence statement is not accurate and  there are restrictions on sharing of data and/or materials, please state these. Please note that we cannot proceed with consideration of your article until this information has been declared.

Please include both an updated Funding Statement and Competing Interests Statement in your cover letter. We will change the online submission form on your behalf. *This has been done as stated above and is in the cover letter.*

7. We note that you have indicated that data from this study are available upon request. PLOS only allows data to be available upon request if there are legal or ethical restrictions on sharing data publicly. For more information on unacceptable data access restrictions, please see <http://journals.plos.org/plosone/s/data-availability#loc-unacceptable-data-access-restrictions>.

In your revised cover letter, please address the following prompts: *Our response is under b) and mentioned in the cover letter.*

a) If there are ethical or legal restrictions on sharing a de-identified data set, please explain them in detail (e.g., data contain potentially sensitive information, data are owned by a third-party organization, etc.) and who has imposed them (e.g., an ethics committee). Please also provide contact information for a data access committee, ethics committee, or other institutional body to which data requests may be sent.

b) If there are no restrictions, please upload the minimal anonymized data set necessary to replicate your study findings as either Supporting Information files or to a stable, public repository and provide us with the relevant URLs, DOIs, or accession numbers. For a list of acceptable repositories, please see <http://journals.plos.org/plosone/s/data-availability#loc-recommended-repositories>.

*The data has been up-loaded and link is provided in the cover letter.*

We will update your Data Availability statement on your behalf to reflect the information you provide.

[Note: HTML markup is below. Please do not edit.]

Reviewers' comments:

Reviewer's Responses to Questions

**Comments to the Author**

1. Is the manuscript technically sound, and do the data support the conclusions?

The manuscript must describe a technically sound piece of scientific research with data that supports the conclusions. Experiments must have been conducted rigorously, with appropriate controls, replication, and sample sizes. The conclusions must be drawn appropriately based on the data presented.

Reviewer #1: No

Reviewer #2: No

2. Has the statistical analysis been performed appropriately and rigorously?

Reviewer #1: No

Reviewer #2: No

3. Have the authors made all data underlying the findings in their manuscript fully available?

The [PLOS Data policy](http://www.plosone.org/static/policies.action#sharing) requires authors to make all data underlying the findings described in their manuscript fully available without restriction, with rare exception (please refer to the Data Availability Statement in the manuscript PDF file). The data should be provided as part of the manuscript or its supporting information, or deposited to a public repository. For example, in addition to summary statistics, the data points behind means, medians and variance measures should be available. If there are restrictions on publicly sharing data—e.g. participant privacy or use of data from a third party—those must be specified.

Reviewer #1: No

Reviewer #2: Yes

4. Is the manuscript presented in an intelligible fashion and written in standard English?

PLOS ONE does not copyedit accepted manuscripts, so the language in submitted articles must be clear, correct, and unambiguous. Any typographical or grammatical errors should be corrected at revision, so please note any specific errors here.

Reviewer #1: No

Reviewer #2: No

5. Review Comments to the Author

Please use the space provided to explain your answers to the questions above. You may also include additional comments for the author, including concerns about dual publication, research ethics, or publication ethics. (Please upload your review as an attachment if it exceeds 20,000 characters)

Reviewer #1: A clinical trial was conducted which aimed to study the impact of consuming fortified rice for six months on nutrient levels and vital health status of migrant workers in Singapore. A high percentage of migrant workers exhibited folate and vitamin B12 deficiencies. Consuming fortified rice increased vitamin, iron, and zinc levels. In the subset of Bangladeshi migrant workers, a reduction in systolic blood pressure was observed from pre to post-intervention.

Major revisions:
1- Thoroughly proofread the manuscript. In many instances, phraseology is non-standard. *The manuscript has been checked for English language and grammar by a native English speaking Australian. As per suggestion, the authors have now edited the manuscript using an editing tool.*
2- An underlying assumption of the chi-square test is independent samples. Thus, it is inappropriate to compare baseline and post-intervention proportions (percentages) using the chi-square test. *We agree with the reviewer; it has been an editorial mistake in the manuscript to mention that a chi-square was used to compare pre- and post-intervention outcomes. Chi-square was never used to assess the statistical significance of any comparison in the statistical analysis. An independent t-test was done as stated in the manuscript. The sentence: “P values for apparent differences between the prevalence rates of deficiency at baseline and endpoint were calculated using a chi-square test” should never have been in the manuscript and is deleted.*
3- Include p-values in the text to support claims of statistical significance.
*We have included p-values where the respective statistical analysis was done. We have given explanations (see point 9 below) why we did not include other p-values.*

Minor revisions:
1- Abstract: The term “constitute” in the second sentence is used inappropriately. *We agree and replaced “constitute” with “are” and edited manuscript accordingly.*
2- Clarify the use of the terms “status” and “concentrations” in the abstract. Perhaps “levels” is a more precise term. *We understand the reviewer’s comment but we have not used “concentrations” in the abstract. We accepted the term “levels” instead of “status” in the abstract and throughout the manuscript for specific micronutrient levels but left the term “health and nutrition status” in the manuscript as we believe this is accepted terminology.*

3- Abstract: Provide percentages of those with nutrient deficiencies. *We agree with the reviewer and have edited the sentence in the manuscript as following: the results show that micronutrient deficiencies were present, especially folate (59% of subjects deficient) and vitamin B12 (7% deficient, 31% marginally deficient).*

4- Line 221: Replace the following two sentences with the suggestion below. “To minimize the inter-individual component covariance, the difference between baseline and endline measurements for each individual survey subject was calculated. P values were calculated using a one-sample t-test comparing the average difference to zero.” Suggested replacement: “Paired t-tests were used to compare baseline to post-intervention measurements.” Revise line 260 accordingly. *We agree with the reviewer and edited the manuscript accordingly.*
5- Normal distribution of data is an underlying assumption of the paired or independent t-test. Indicate if the data was checked for normality prior to applying these tests. *Yes - the data was assessed (SPSS output) for normality of the distribution of all the change in indicators; all the indicators were sufficiently close to normally distributed to allow the use of the paired t-test. We have edited the manuscript accordingly.*
5- The term “endpoint” is unclear. Perhaps "post-intervention" is better. *We have accepted the reviewer’s suggestion and have exchanged throughout the manuscript the term “endpoint” with “post-intervention”.*

6- Line 253: Revise: When considering the entire sample, the vital signs of the workers did not change significantly from baseline to post-intervention. *Revision accepted and manuscript edited accordingly.*
7- Line 255: Revise to: increase in the hemoglobin, ferrin, zinc, and vitamin B12 levels. *Revision accepted and manuscript edited accordingly.*
8- P-values never equal zero. Express small p-values as p < 0.001. *We agree with the reviewer and have corrected in Table 2 to p < 0.001 which is the conventional way to display very small probabilities.*
9- Lines 263, 266, 269, 270, 271: Within the text, provide the p-values to support the claims. *We do not show the p-values because comparison of categorical variables between baseline and endline is a much less powerful analysis and is rendered superfluous by the inclusion of the analysis of the indicators as continuous variables with p-values determined by the paired t-test. No statistical analysis was done on dichotomized variables (prevalence data) as it would lead to considerable loss of power; in addition, dichotomizing continuous variables is inadvisable. However, in response to the reviewer’s comment, we edited those lines in the manuscript to make less strong statements.*
10- Throughout the manuscript, provide the frequencies that correspond to the percentages. *Accepted and edited accordingly in the manuscript Tables 1-3 with frequencies that correspond to the percentages.*

11- State and justify the study’s target sample size with a pre-study statistical power calculation.
The power calculation should include: (1) the estimated outcomes in each group; (2) the α (type I) error level; (3) the statistical power (or the β (type II) error level); (4) the target sample size and (5) for continuous outcomes, the standard deviation of the measurements.

*A sample size of 180 participants with a maximum anticipated subject attrition of 22% was estimated based on the following: a paired t-test, a power of 80% to achieve significance on all primary endpoints simultaneously (95% power for each primary endpoint individually) and a two-sided alpha of 5% (overall), and an effect size of ≥0.30 for all parameters. The study suffered from high dropout rates; first, with 40 of the original 180 workers who did not report for screening, and second, an additional 40 of the 140 remaining participants who either left the study or declined involvement in the final study visit. The aimed sample size of 140 for an overall power of 80% was not achieved, and therefore, the study might be underpowered to pick up significant effects. We have edited the manuscript in the statistical section accordingly.*

Reviewer #2: Abstract - Need data to be showed to indicate the improvement of nutrtional status (as mentioned) *Agree with reviewer and the abstract has been edited to include results (same response as to reviewer #1)*
Introduction - 1.Explain the relationship between nutrient deficiencies in relation to homocystein and blood pressure. *We agree with the reviewer and have added a sentence into the introduction to explain this relationship.*
2. Are there the previous studies / data of nutritional status of male worker in Singapore ? *To our knowledge, there are no scientific published studies on nutritional status of male migrant workers in Singapore. This is also the strength of our study as we believe we are the first to publish in a peer-reviewed journal on this subject as only limited anecdotal information is available. We refer to this at the start of the Discussion section.*

*.*why is important compare to other group of population? *We are not sure to understand this comment but we believe that every person has the right to have a good nutrition and health status; we do not compare our subjects to a control group as we have no control but we compare the micronutrient and health status to global references of a healthy population.*
Method 1. Expain about how to select the study site ? and sample size calculation ? *The study site was provided by a Singaporean company who employed migrant workers in their construction sites. The construction sites were selected by the company and were the same throughout the study. A sample size of 180 participants with a maximum anticipated subject attrition of 22% was estimated based on the following: a paired t-test, a power of 80% to achieve significance on all primary endpoints simultaneously (95% power for each primary endpoint individually) and a two-sided alpha of 5% (overall), and an effect size of ≥0.30 for all parameters. The study suffered from high dropout rates; first, with 40 of the original 180 workers who did not report for screening, and second, an additional 40 of the 140 remaining participants who either left the study or declined involvement in the final study visit. The aimed sample size of 140 for an overall power of 80% was not achieved, and therefore, the study might be underpowered to pick up significant effects. We have edited the manuscript in the statistical section accordingly (same response as to reviewer #1).*
2. Participant was given 500 g cooked rice per day or per meal (line no .119,126) *500 g cooked rice was given per meal; we edited the manuscript accordingly*
3. Line 181-184 ...100 g uncooked rice equal to how much for cooked rice? *100 g uncooked rice transforms itself to approximately 300 g cooked rice; the quantities of rice per meal were decided by the caterers and in agreement with the workers and their cultural food habit*
4. Explain about the nutrients level which participant was recieved form fortified rice / day *The calculations are based on the following assumptions: 100 g uncooked rice transforms into 300 g cooked rice; 500 g cooked rice is then approximately 167 g uncooked rice. The nutrient level for twice a meal with each 500 g cooked rice would be: 500 µg vitamin A; 1.6 mg B1; 23.4 mg B3; 20 mg B6; 3.34 µg B12; 434 µg Folic Acid; 13.36 mg Fe; 20 mg Zn. These nutrient levels are in line with the daily nutrient intake recommendation and do not exceed the upper limit levels. We added the following sentence into the manuscript: Consuming their daily meals, the workers receive the following nutrient support: 500 µg vitamin A; 1.6 mg B1; 23.4 mg B3; 20 mg B6; 3.34 µg B12; 434 µg Folic Acid; 13.36 mg Fe; 20 mg Zn. This is based on 500 g cooked rice (per meal) to be approximately 167 g of uncooked rice (per meal).*
5. Blood collection for serum Zn need to be collected, prepared with special condition to prevent contatimination e.g. all supplied used for blood collection need to be the trace element free apparatus (<https://www.izincg.org/new-blog-1/2021/1/10/comparison-of-laboratory-instrument-types-for-analysis-of-plasma-or-serum-zinc-concentration>) ... For my opinion, results of serum Zn may not be reliable. *As much as we agree with the reviewer, the tubes that were used were Royal Blue Cap (Red Band) trace element free. Therefore, there is probably no concern. We have edited a sentence into the manuscript under the Methods section which states that the tubes were trace element free and that all laboratory measurements were done according to international standards.*
6. How to control confounding factors e.g. life stye, diet, genetic *We acknowledge the weakness of the study; the study is an open label “effectiveness study” which is a pragmatic trial measuring the degree of beneficial effect under “real world” conditions and not a randomized controlled trial with a control group. Therefore it was not possible to adjust for existing confounding variables that might have changed over the time during this study (like season, diet or life style). We have added a sentence under the section of the limitations of the study in the manuscript.*
7. What 's the QC procedure / or data of refference materials for the biochemical data. *The Innovative Lab, Singapore, which did the analysis used stringent quality control procedures and laboratory methods that meet international standards. See also their catalogue:* [*Innovative-Diagnostics-Service-Catalogue-2017.pdf (innovativelab.com.sg)*](https://www.innovativelab.com.sg/wp-content/uploads/2019/08/Innovative-Diagnostics-Service-Catalogue-2017.pdf) *We have edited a sentence into the manuscript stating that all laboratory measurements were done according to international standards.*

8. Why the author used independent t-test ? (since participants are the same group) *The independent t-test was used to compare different subgroups at baseline, i.e. comparing Indians to Bangladeshis. The Indian subgroup is an independent subsample of our population just like the Bangladeshis, so independent t-test is an appropriate measure of precision for differences found between subgroups.*

Results
1. Data need to be revised if statistical analysis would change *Indeed, the data would need to be revised if we would change the statistical analysis. However, we are not changing the statistical analysis as we did the right test (independent t-test); we had mentioned in the manuscript to have done a chi-square test but this had been an editorial mistake (we have now corrected it in the manuscript) as we did not do such a test, only the t-test. Hence, we leave the data as they are.*
2. Restults of serum Zn should be removed since they not reliable *Please see our answer above under point 5. We also believe to remove the Zn results would be unethical as these data were collected.*
3. I would suggest that the data should be presented for both pre - and post-in the same table / figure
and the text need to be changed accordingly *We agree with the reviewer’s comment; nevertheless Table 3 shows already baseline and post-intervention results for the number of workers that finished the study from enrolment to post-intervention (n=100 and n=18 for homocysteinemia). Table 1 has the purpose of showing the profile of all the workers (N=133) enrolled at baseline as we believe it is important to describe that the worker population was diagnosed with substantial micronutrient deficiencies.*
4. The text need to be re-write to be more clearly and logically manner (make it mor easy to understand) *We have edited the manuscript.*

Discussion and conclusion
1. Line 287. What's the indicator which show " micronutrient deficiency was commonly reported amongst the male migrant worker" *We acknowledge that this sentence might be confusing as micronutrient deficiency is usually not reported but discovered by examination or diagnostic testing; we edited the manuscript accordingly and used the word “diagnosed” instead of “reported”. Furthermore to the reviewer’s question on indicator, there is no single indicator for general micronutrient deficiency and therefore, several biomarkers indicate the presence of specific deficiencies, including deficiencies of folate, vitamin B12 and others as used in our study.* line 374 "This study has shown that hidden hunger is highly prevalent" in this study *Micronutrient deficiency is also called hidden hunger; therefore, the same comments as above apply and no single indicator exists to assess hidden hunger.*

2. Any other underlining cause or confounding factor contributed in the results e.g. life stye, diet, genetic *We acknowledge the reviewer’s comment; we have edited the manuscript accordingly and have inserted in the section “limitations of the study” the point that the absence of a control group means that it was not possible to adjust for existing confounding variables such as life style, diet, genetic, etc.*

6. PLOS authors have the option to publish the peer review history of their article ([what does this mean?](https://journals.plos.org/plosone/s/editorial-and-peer-review-process#loc-peer-review-history)). If published, this will include your full peer review and any attached files.


If you choose “no”, your identity will remain anonymous but your review may still be made public.


**Do you want your identity to be public for this peer review?** For information about this choice, including consent withdrawal, please see our [Privacy Policy](https://www.plos.org/privacy-policy).

Reviewer #1: No

Reviewer #2: No

[NOTE: If reviewer comments were submitted as an attachment file, they will be attached to this email and accessible via the submission site. Please log into your account, locate the manuscript record, and check for the action link "View Attachments". If this link does not appear, there are no attachment files.]

While revising your submission, please upload your figure files to the Preflight Analysis and Conversion Engine (PACE) digital diagnostic tool, <https://pacev2.apexcovantage.com/>. PACE helps ensure that figures meet PLOS requirements. To use PACE, you must first register as a user. Registration is free. Then, login and navigate to the UPLOAD tab, where you will find detailed instructions on how to use the tool. If you encounter any issues or have any questions when using PACE, please email PLOS at [figures@plos.org](mailto:figures@plos.org). Please note that Supporting Information files do not need this step.

*In compliance with data protection regulations, you may request that we remove your personal registration details at any time.* [*(Remove my information/details)*](https://www.editorialmanager.com/pone/login.asp?a=r)*. Please contact the publication office if you have any questions.*
