# Supplementary material for: Optimizing health and nutrition status of migrant construction workers consuming multiple micronutrient fortified rice in Singapore
Source: PLoS One. 2023 Jun 1;18(6):e0285708. doi: 10.1371/journal.pone.0285708 (PMC10234550; doi:10.1371/journal.pone.0285708)
Supplement: Attachment — Submitted filename: Response to Reviewers July 30.docx [file pone.0285708.s008.docx]

Dear George Vousden
Deputy Editor in Chief

We have addressed your comment in italics below in this message; we have also accordingly revised the manuscript.

Kind regards

Regina Moench Pfanner

**From:** em.pone.0.7cbe94.60095dba@editorialmanager.com [mailto:em.pone.0.7cbe94.60095dba@editorialmanager.com] **On Behalf Of** PLOS ONE
**Sent:** Tuesday, 19 July 2022 3:43 AM
**To:** Regina Moench Pfanner <regina@ibn-360.com>
**Subject:** PLOS ONE Decision: Revision required [PONE-D-21-12903R1] - [EMID:4b7e117b38575169]

PONE-D-21-12903R1

Optimizing health and nutrition status of migrant construction workers consuming multiple micronutrient fortified rice in Singapore

PLOS ONE

Dear Dr. Moench Pfanner,

Thank you for submitting your manuscript to PLOS ONE. After careful consideration, we feel that it has merit but does not fully meet PLOS ONE’s publication criteria as it currently stands. Therefore, we invite you to submit a revised version of the manuscript that addresses the points raised during the review process.

The concerns noted by the reviewers have been addressed. However, during my own review of the manuscript I noted some potential ethical concerns that should be addressed before the manuscript is published:

Your clinical trial was conducted in a dormitory. Your manuscript indicates that 140 of the ~180 members of the dormitory consented to participate in the study. Please update your manuscript to indicate what food individuals who did not consent to take part received. Did they also receive the fortified rice? If individuals that did not consent to take part were given the fortified rice, despite not consenting to take part in the study, please discuss how the associated ethical concerns with this were mitigated. *The workers who were not participating in the study were not provided the fortified rice. The employer/dormitory does not provide food as it is not part of the Ministry of Manpower requirement for these employers to provide food. It is up to the workers to arrange food for themselves. Typically, they would arrange with a catering company of their own choosing. In our study, the dormitory/site manager organized the meals for those workers who consented to participate in the study. We have inserted the following sentence into the manuscript: The workers who did not consent to the study, did not receive the fortified rice and organized their meals by their own choice.*

Please submit your revised manuscript by Aug 29 2022 11:59PM. If you will need more time than this to complete your revisions, please reply to this message or contact the journal office at [plosone@plos.org](mailto:plosone@plos.org). When you're ready to submit your revision, log on to <https://www.editorialmanager.com/pone/> and select the 'Submissions Needing Revision' folder to locate your manuscript file.

Please include the following items when submitting your revised manuscript:

- A rebuttal letter that responds to each point raised by the academic editor and reviewer(s). You should upload this letter as a separate file labeled 'Response to Reviewers'.
- A marked-up copy of your manuscript that highlights changes made to the original version. You should upload this as a separate file labeled 'Revised Manuscript with Track Changes'.
- An unmarked version of your revised paper without tracked changes. You should upload this as a separate file labeled 'Manuscript'.

If you would like to make changes to your financial disclosure, please include your updated statement in your cover letter. Guidelines for resubmitting your figure files are available below the reviewer comments at the end of this letter.

If applicable, we recommend that you deposit your laboratory protocols in protocols.io to enhance the reproducibility of your results. Protocols.io assigns your protocol its own identifier (DOI) so that it can be cited independently in the future. For instructions see: <https://journals.plos.org/plosone/s/submission-guidelines#loc-laboratory-protocols>. Additionally, PLOS ONE offers an option for publishing peer-reviewed Lab Protocol articles, which describe protocols hosted on protocols.io. Read more information on sharing protocols at <https://plos.org/protocols?utm_medium=editorial-email&utm_source=authorletters&utm_campaign=protocols>.

We look forward to receiving your revised manuscript.

Kind regards,

George Vousden
Deputy Editor in Chief
PLOS ONE

[Note: HTML markup is below. Please do not edit.]

Reviewers' comments:

Reviewer's Responses to Questions

**Comments to the Author**

1. If the authors have adequately addressed your comments raised in a previous round of review and you feel that this manuscript is now acceptable for publication, you may indicate that here to bypass the “Comments to the Author” section, enter your conflict of interest statement in the “Confidential to Editor” section, and submit your "Accept" recommendation.

Reviewer #1: (No Response)

2. Is the manuscript technically sound, and do the data support the conclusions?

The manuscript must describe a technically sound piece of scientific research with data that supports the conclusions. Experiments must have been conducted rigorously, with appropriate controls, replication, and sample sizes. The conclusions must be drawn appropriately based on the data presented.

Reviewer #1: Yes

3. Has the statistical analysis been performed appropriately and rigorously?

Reviewer #1: Yes

4. Have the authors made all data underlying the findings in their manuscript fully available?

The [PLOS Data policy](http://www.plosone.org/static/policies.action#sharing) requires authors to make all data underlying the findings described in their manuscript fully available without restriction, with rare exception (please refer to the Data Availability Statement in the manuscript PDF file). The data should be provided as part of the manuscript or its supporting information, or deposited to a public repository. For example, in addition to summary statistics, the data points behind means, medians and variance measures should be available. If there are restrictions on publicly sharing data—e.g. participant privacy or use of data from a third party—those must be specified.

Reviewer #1: Yes

5. Is the manuscript presented in an intelligible fashion and written in standard English?

PLOS ONE does not copyedit accepted manuscripts, so the language in submitted articles must be clear, correct, and unambiguous. Any typographical or grammatical errors should be corrected at revision, so please note any specific errors here.

Reviewer #1: Yes

6. Review Comments to the Author

Please use the space provided to explain your answers to the questions above. You may also include additional comments for the author, including concerns about dual publication, research ethics, or publication ethics. (Please upload your review as an attachment if it exceeds 20,000 characters)

Reviewer #1: (No Response)

7. PLOS authors have the option to publish the peer review history of their article ([what does this mean?](https://journals.plos.org/plosone/s/editorial-and-peer-review-process#loc-peer-review-history)). If published, this will include your full peer review and any attached files.


If you choose “no”, your identity will remain anonymous but your review may still be made public.


**Do you want your identity to be public for this peer review?** For information about this choice, including consent withdrawal, please see our [Privacy Policy](https://www.plos.org/privacy-policy).

Reviewer #1: No

[NOTE: If reviewer comments were submitted as an attachment file, they will be attached to this email and accessible via the submission site. Please log into your account, locate the manuscript record, and check for the action link "View Attachments". If this link does not appear, there are no attachment files.]

While revising your submission, please upload your figure files to the Preflight Analysis and Conversion Engine (PACE) digital diagnostic tool, <https://pacev2.apexcovantage.com/>. PACE helps ensure that figures meet PLOS requirements. To use PACE, you must first register as a user. Registration is free. Then, login and navigate to the UPLOAD tab, where you will find detailed instructions on how to use the tool. If you encounter any issues or have any questions when using PACE, please email PLOS at [figures@plos.org](mailto:figures@plos.org). Please note that Supporting Information files do not need this step.

*In compliance with data protection regulations, you may request that we remove your personal registration details at any time.* [*(Remove my information/details)*](https://www.editorialmanager.com/pone/login.asp?a=r)*. Please contact the publication office if you have any questions.*
