# Supplementary material for: Optimizing health and nutrition status of migrant construction workers consuming multiple micronutrient fortified rice in Singapore
Source: PLoS One. 2023 Jun 1;18(6):e0285708. doi: 10.1371/journal.pone.0285708 (PMC10234550; doi:10.1371/journal.pone.0285708)
Supplement: Attachment — Submitted filename: Response to Reviewer Letter 31 October 2022.docx [file pone.0285708.s009.docx]

REBUTTAL LETTER

Dear Editors,

**We have addressed all your comments and have inserted our responses in italics into your comments below; we have accordingly edited the manuscript, the TREND statement checklist and cover letter as per below requirements.**

PONE-D-21-12903R2

Optimizing health and nutrition status of migrant construction workers consuming multiple micronutrient fortified rice in Singapore

PLOS ONE

Dear Dr. Moench Pfanner,

Thank you for submitting your manuscript to PLOS ONE. After careful consideration, we feel that it has merit but does not fully meet PLOS ONE’s publication criteria as it currently stands. Therefore, we invite you to submit a revised version of the manuscript that addresses the points raised during the review process.

Please see below for additional comments from the editor that require a response.

Please submit your revised manuscript by Nov 12 2022 11:59PM. If you will need more time than this to complete your revisions, please reply to this message or contact the journal office at [plosone@plos.org](mailto:plosone@plos.org). When you're ready to submit your revision, log on to <https://www.editorialmanager.com/pone/> and select the 'Submissions Needing Revision' folder to locate your manuscript file.

Please include the following items when submitting your revised manuscript:

- A rebuttal letter that responds to each point raised by the academic editor and reviewer(s). You should upload this letter as a separate file labeled 'Response to Reviewers'.
- A marked-up copy of your manuscript that highlights changes made to the original version. You should upload this as a separate file labeled 'Revised Manuscript with Track Changes'.
- An unmarked version of your revised paper without tracked changes. You should upload this as a separate file labeled 'Manuscript'.

If you would like to make changes to your financial disclosure, please include your updated statement in your cover letter. Guidelines for resubmitting your figure files are available below the reviewer comments at the end of this letter.

If applicable, we recommend that you deposit your laboratory protocols in protocols.io to enhance the reproducibility of your results. Protocols.io assigns your protocol its own identifier (DOI) so that it can be cited independently in the future. For instructions see: <https://journals.plos.org/plosone/s/submission-guidelines#loc-laboratory-protocols>. Additionally, PLOS ONE offers an option for publishing peer-reviewed Lab Protocol articles, which describe protocols hosted on protocols.io. Read more information on sharing protocols at <https://plos.org/protocols?utm_medium=editorial-email&utm_source=authorletters&utm_campaign=protocols>.

We look forward to receiving your revised manuscript.

Kind regards,

Callam Davidson
Editorial Office
PLOS ONE

Additional Editor Comments:

The following content currently found in your Financial Disclosure should be relocated to your Competing Interests: ‘RMP works at ibn360 and received financial support by the funder for consultancy. KM was affiliated at 45RICE at the time of the study, a social for-profit business by selling a product or service to the public and acquiring funds through sales. FH was employed by DNP at the time of the study.’ *We have edited the Cover Letter accordingly.*

Please confirm whether the informed consent was written or verbal, and include this information in your Methods. *Informed consent was written and we edited the manuscript accordingly.*

Line 77: Please update ‘1960’s’ to ‘1960s’. *Edited accordingly in manuscript.*

Line 121: Please update ‘andS2’ to ‘and S2’. *Edited accordingly in manuscript.*

Your TREND checklist is missing details of how sample size was calculated, however this information is contained within the manuscript (at lines 238-245). Please update your checklist to reflect this. *We acknowledge this comment and have edited the TREND statement checklist accordingly with a checkbox and have inserted the text into the Point “Sample Size”(7) in the checklist.*

Please also cite your TREND checklist in the Methods. *We have edited the manuscript accordingly and have added the following sentence into the methods section: The TREND statement checklist was established to allow transparent reporting of the study.*

Your protocol mentions adverse event monitoring, but this is not reported in the manuscript – please update your manuscript and TREND checklist to include this information. *We acknowledge this comment and yes, indeed we have collected data on AEs and have adjusted the TREND statement checklist with a checkbox in “Adverse events” (19) and have adjusted the Methods section in the manuscript to describe what was done; and have also adjusted the Results section in the manuscript with a descriptive summary and of the number and type of the AEs. No statistical analysis was planned for or performed on the AEs. Manuscript was edited accordingly.*

There is a discrepancy between the protocol and clinical trail registry information at <https://clinicaltrials.gov/ct2/show/NCT04343508>. Your primary and secondary endpoints do not match. Please explain this discrepancy and ensure the correct information is reported in the manuscript. Primary and secondary outcomes ought to be clearly demarcated in the Abstract, Methods, and the Results. *The study protocol defined the multiple nutritional biomarkers as co-primary endpoints simultaneously* *for which the study was powered (80%). This is an accepted approach and was also approved in the protocol by the Ethics Committee. When registering the protocol at the ClinicalTrials.gov website, the organization did not accept co-primary endpoints the reason why only one of the biomarkers was selected as primary endpoint, even though the study was designed to analyse all co-primary endpoints simultaneously. We also discuss the totality of nutritional biomarkers as an indication of nutritional status in our paper.*

Line 204: Please state the number of randomly selected workers from whom additional blood was planned for collection (I believe this information is located at line 270). *We have edited the manuscript accordingly and have added the number of 53 as the subset were 53 workers.*

Line 245: The underpowered nature of the study needs to be stated as a limitation in your Discussion. *We have edited the manuscript accordingly and have added a sentence to the limitation section in our discussion.*

Line 248: Please include the rationale for this deviation from the protocol. *In the study protocol, both a per-protocol and an intention-to-treat analysis were planned, with the latter being considered as primary analysis. However, an intention-to-treat and not a per-protocol analysis was performed, since little difference between the two analyses was expected; the workers ate all their meals in the dining facilities, so compliance with consumption of the fortified foodstuffs was relatively high. However, there were many study subjects who lacked the single follow-up data collection because they were on home leave. As a result, no outcomes can be measured in these study subjects. Therefore, there was either excellent compliance or the volunteer could not be included in the analysis because of a lack of data. As a result, there is little difference between intention-to-treat and per-protocol analyses.*

Line 298: Please updated to ‘statistically significant’. *We have edited the manuscript accordingly.*

Line 305: Please justify the decision not to perform statistical analysis. I do not feel that the data presented in Table 3 necessarily support the statements on lines 304-307. *These categories are only descriptively reported as the study was not designed to detect changes and they were not part of the planned statistical analysis. We have adjusted the statements in previously lines 304-307 which are now lines 332-338 in manuscript with track changes.*

Table 3: Please consider including gridlines to make this table easier to read. *We agree with the reviewer and have included gridlines in all tables to improve readability.*

Line 320: Please avoid overstating conclusions based on the findings presented – please temper the statement ‘an effective strategy’ by adding ‘potentially’, or similar. *We acknowledge this comment and have edited the sentence accordingly in the manuscript.*

Line 411: In a similar vein, please temper or remove the statement ‘multi-micronutrient-fortified rice may be the ideal vehicle to optimize the health status’, as the findings from this study cannot support this claim. *We acknowledge this comment and have edited the sentence accordingly in the manuscript.*

Line 426: From the information presented, it appears BAW may qualify for authorship based on ICMJE criteria. Please review <https://journals.plos.org/plosone/s/authorship> and determine whether BAW ought to be included as an author.  *We were aware of this point and BAW was offered to co-author, but was not interested in co-authorship.*

[Note: HTML markup is below. Please do not edit.]

Reviewers' comments:

Reviewer's Responses to Questions

**Comments to the Author**

1. If the authors have adequately addressed your comments raised in a previous round of review and you feel that this manuscript is now acceptable for publication, you may indicate that here to bypass the “Comments to the Author” section, enter your conflict of interest statement in the “Confidential to Editor” section, and submit your "Accept" recommendation.

Reviewer #1: All comments have been addressed

2. Is the manuscript technically sound, and do the data support the conclusions?

The manuscript must describe a technically sound piece of scientific research with data that supports the conclusions. Experiments must have been conducted rigorously, with appropriate controls, replication, and sample sizes. The conclusions must be drawn appropriately based on the data presented.

Reviewer #1: (No Response)

3. Has the statistical analysis been performed appropriately and rigorously?

Reviewer #1: (No Response)

4. Have the authors made all data underlying the findings in their manuscript fully available?

The [PLOS Data policy](http://www.plosone.org/static/policies.action#sharing) requires authors to make all data underlying the findings described in their manuscript fully available without restriction, with rare exception (please refer to the Data Availability Statement in the manuscript PDF file). The data should be provided as part of the manuscript or its supporting information, or deposited to a public repository. For example, in addition to summary statistics, the data points behind means, medians and variance measures should be available. If there are restrictions on publicly sharing data—e.g. participant privacy or use of data from a third party—those must be specified.

Reviewer #1: (No Response)

5. Is the manuscript presented in an intelligible fashion and written in standard English?

PLOS ONE does not copyedit accepted manuscripts, so the language in submitted articles must be clear, correct, and unambiguous. Any typographical or grammatical errors should be corrected at revision, so please note any specific errors here.

Reviewer #1: (No Response)

6. Review Comments to the Author

Please use the space provided to explain your answers to the questions above. You may also include additional comments for the author, including concerns about dual publication, research ethics, or publication ethics. (Please upload your review as an attachment if it exceeds 20,000 characters)

Reviewer #1: (No Response)

7. PLOS authors have the option to publish the peer review history of their article ([what does this mean?](https://journals.plos.org/plosone/s/editorial-and-peer-review-process#loc-peer-review-history)). If published, this will include your full peer review and any attached files.


If you choose “no”, your identity will remain anonymous but your review may still be made public.


**Do you want your identity to be public for this peer review?** For information about this choice, including consent withdrawal, please see our [Privacy Policy](https://www.plos.org/privacy-policy).

Reviewer #1: No

[NOTE: If reviewer comments were submitted as an attachment file, they will be attached to this email and accessible via the submission site. Please log into your account, locate the manuscript record, and check for the action link "View Attachments". If this link does not appear, there are no attachment files.]

While revising your submission, please upload your figure files to the Preflight Analysis and Conversion Engine (PACE) digital diagnostic tool, <https://pacev2.apexcovantage.com/>. PACE helps ensure that figures meet PLOS requirements. To use PACE, you must first register as a user. Registration is free. Then, login and navigate to the UPLOAD tab, where you will find detailed instructions on how to use the tool. If you encounter any issues or have any questions when using PACE, please email PLOS at [figures@plos.org](mailto:figures@plos.org). Please note that Supporting Information files do not need this step.

*In compliance with data protection regulations, you may request that we remove your personal registration details at any time.* [*(Remove my information/details)*](https://www.editorialmanager.com/pone/login.asp?a=r)*. Please contact the publication office if you have any questions.*
